# Empirical phenotyping and genome-wide association study reveal the association of panicle architecture with yield in *Chenopodium quinoa*

**DOI:** 10.3389/fmicb.2024.1349239

**Published:** 2024-03-18

**Authors:** Zakia Habib, Siddra Ijaz, Imran Ul Haq, Abeer Hashem, Graciela Dolores Avila-Quezada, Elsayed Fathi Abd_Allah, Nasir Ahmad Khan

**Affiliations:** ^1^Centre of Agricultural Biochemistry and Biotechnology (CABB), University of Agriculture, Faisalabad, Pakistan; ^2^Department of Plant Pathology, University of Agriculture, Faisalabad, Pakistan; ^3^Botany and Microbiology Department, College of Science, King Saud University, Riyadh, Saudi Arabia; ^4^Facultad de Ciencias Agrotecnológicas, Universidad Autónoma de Chihuahua, Chihuahua, Mexico; ^5^Plant Production Department, College of Food and Agricultural Sciences, King Saud University, Riyadh, Saudi Arabia

**Keywords:** heterogeneous germplasm, phenotypic characterization, ecological genomics, GWAS, phenomics

## Abstract

*Chenopodium quinoa* manifests adaptability to grow under varying agro-climatic scenarios. Assessing quinoa germplasm’s phenotypic and genetic variability is a prerequisite for introducing it as a potential candidate in cropping systems. Adaptability is the basic outcome of ecological genomics of crop plants. Adaptive variation predicted with a genome-wide association study provides a valuable basis for marker-assisted breeding. Hence, a panel of 72 quinoa plants was phenotyped for agro morphological attributes and association-mapping for distinct imperative agronomic traits. Inter simple sequence repeat (ISSR) markers were employed to assess genetic relatedness and population structure. Heatmap analysis showed three genotypes were early maturing, and six genotypes were attributed for highest yield. The SD-121-07 exhibited highest yield per plant possessing green, glomerulate shaped, compact density panicle with less leaves. However, SJrecm-03 yielded less exhibiting pink, intermediate shape, intermediate density panicles with less leaves. The phenotyping revealed strong correlation of panicle architecture with yield in quinoa. A genome-wide association study unraveled the associations between ISSR makers and agro-morphological traits. Mixed linear modes analysis yielded nine markers associated with eight traits at *p* ≤ 0.01. Moreover, ISSR markers significantly associated with panicle shape and leafiness were also associated with yield per plant. These findings contribute to the provision of authenticity for marker-assisted selection that ultimately would support quinoa breeding programs.

## Introduction

*Chenopodium quinoa*, commonly known as quinoa, is an important pseudocereal originally cultivated in Andean regions of South America in the ancient era (Mad et al., 2006; Vega-Gálvez et al., 2010). Quinoa is primarily grown in Peru, Chile, Bolivia, Ecuador, Colombia, and Argentina, but later on, it has been familiarized in other states, such as North America, Europe, Asia, and Africa, with greater yields (Vilcacundo and Hernández-Ledesma, 2017; Gomez-Pando et al., 2019). It belongs to the family Amaranthaceae and is considered a superfood because of its high nutritional value and ability to withstand various agroecological conditions (García-Parra et al., 2020). Globally, it has gained more attention because of its gluten-free grains (Pathan and Siddiqui, 2022) and exceptional nutritional properties, including high protein content and balanced essential amino acids, lipids, and vitamins (Carciochi et al., 2016; Vilcacundo and Hernández-Ledesma, 2017). Moreover, it possesses terpenoids, flavonoids, phenolic acids, steroids, and nitrogen-containing compounds that have antidiabetic, antimicrobial, anti-inflammatory, anticancer, and immunoregulatory properties (Burrieza et al., 2019; Pereira et al., 2019, 2020). As an environment-resilient crop (Ruiz et al., 2014), it can grow in hot, arid deserts to tropical climates with approximately 88% relative humidity, −8 to 40°C temperature, and from sea level to high altitudes (Tapia, 2015). Furthermore, its ability to grow in soils with pH levels between 4.5 and 9.06 is remarkable, making it suitable for sodic and alkaline soils (Jacobsen, 2003). Thus, this adaptability makes quinoa a promising substitute for traditional crops in adverse climatic scenarios (Sosa-Zuniga et al., 2017).

Plant growth and development have been progressively studied because of climatic modifications affecting natural ecosystems and agricultural production (Korres et al., 2016). Amaranth, buckwheat, chia, and quinoa are underutilized due to the impact of their introduction on crops, including rice, wheat, soybeans, and barley (García-Parra et al., 2020). However, underutilized crops are crucial parts of the confined agricultural system and include a range of historically used crops that may be able to adapt climate change, have therapeutic characteristics, and be developed into functional foods (Ruiz et al., 2014). Among them, quinoa exhibits high phenotypic variability that is easily distinguished by the plant’s pigmentation, seeds and inflorescence type, the density of the panicles, variety of grain size and shape, and resistance to adverse environments such as drought, frost, excessive humidity, and various diseases (Babar et al., 2021; Manjarres-Hernández et al., 2021).

The Food and Agriculture Organization of the United Nations (FAO) reported that between 2000 and 2019, there was a considerable rise in the land area allocated to quinoa farming around the world, mostly in Bolivia and Peru, with increases of 36% and 72%, respectively (Jaikishun et al., 2019; García-Parra et al., 2020). The South American regions, namely Peru, Ecuador, and Bolivia, are the leading quinoa-producing countries that account for 80% of global production (Bazile and Baudron, 2015; Bazile et al., 2016). However, data on various quinoa accessions’ morphological characterization and productivity are limited. Therefore, morphological characterization using qualitative and quantitative traits of different quinoa accessions is crucial to increase crop productivity under various environmental conditions to meet global food demand. Furthermore, recruiting certified seed registration procedures will make it easier to distinguish between accessions, establish core collections, spot any duplicates in seed collections, and improve genotype selection for conservation and breeding programs (Coronado et al., 2020). The present study aimed at the phenotypic characterization of *C. quinoa* germplasm of diverse geographical regions using qualitative and quantitative descriptors to assess their adaptability and ecological genomics to agro-ecological zone of Pakistan for crop diversification.

Genetic characterization is an integral measure in crop improvement, providing valuable information for selection and breeding programs (Fuentes et al., 2009). Moreover, it helps researchers develop new cultivars with desired traits, including improved quality and yield. Assessing genetic diversity is crucial in integrating novel traits necessary for plant production in adverse climatic conditions (El-Harty et al., 2021). According to Zhang et al. (2017), breeding to advance quinoa genotypes is limited due to the paucity of genetic and genomic data on this crop. So, understanding the plant’s genetic diversity, population structure, and genomic variation is highly desirable (Zhang et al., 2017; Ijaz et al., 2018). Genetic markers have been widely used for germplasm conservation and developing core collections without the influence of environmental variables (Abd El-Moneim et al., 2021). Several markers have been documented to scrutinize quinoa genotypes’ genetic variability and phylogenetic associations (Fuentes et al., 2009; Zhang et al., 2017; Abd El-Moneim et al., 2021; El-Harty et al., 2021). Inter simple sequence repeat (ISSR) markers are powerful tools in genetic diversity analysis, as these are considered arbitrarily amplified dominant markers, cost-effective, and actively detect polymorphisms (Thakur et al., 2018; Haq and Ijaz, 2019). These markers require a minute quantity of DNA and do not necessitate prior knowledge of DNA sequence. ISSR primers are designed from SSR (simple sequence repeat) motifs and can be applicable in a wide range of plant species possessing a sufficient number and distribution of these motifs in the genome (Ibrahim et al., 2019; Ijaz et al., 2019).

Molecular mapping helps the genomic-level detection of agromorphological traits by employing molecular markers close to the targeted trait and, therefore, is used in marker-assisted selection (Pandurangan et al., 2021). Molecular markers based on linkage disequilibrium could upturn the efficiency of identifying linked loci to desirable traits within a diverse population. The underlying association mapping principle is linkage disequilibrium that identifies the non-random alleles association at various genetic loci (Flint-Garcia et al., 2003). Consequently, association mapping employs LD to pinpoint the association between genetic polymorphism and variation in phenotypic traits (Flint-Garcia et al., 2003; Zondervan and Cardon, 2004). The present study aimed at the pragmatic phenotyping using qualitative and quantitative descriptors and genome-wide association study in quinoa accessions for distinct imperative agronomic traits.

## Materials and methods

### Germplasm collection

Forty-three quinoa accessions imported from King Abdullah University Science Technology (KAUST) were collected from the Department of Agronomy, University of Agriculture, Faisalabad, Pakistan (Supplementary Table 1). These accessions were geographically different origin including Peru, Bolivia, and USA.

### Phenotyping

#### Field experiment

The association panel of *C. quinoa* accessions was grown at three different locations of University of Agriculture, Faisalabad, Pakistan for 3 consecutive years. As heterogeneity is a strong bottleneck for genetic study, each accession was sown in an isolated plot in the field to ensure the homogeneity of the collected seed germplasm. For the genetic analysis, heterogeneity was scored for each plot within a population of each accession. Following the phenotypic cards developed by Stanschewski et al. (2021), plots were excluded for further analysis where >50% of the plants within a plot were segregating, i.e., when the accession’s main phenotype was not observable. Thirty plants for each accession were sown with 15 cm plant spacing in each plot.

#### Experiment layout

The experiment was designed in a randomized complete block design (RCBD) with split-plot arrangements on a total area of 71 ft^2^. The soil was prepared with two plowings (30 cm depth) followed by planking to conserve moisture content for seed emergence. Ridges of 30 cm height were prepared with 75 cm spacing between each ridge. Seeds were sown in a plot manually by dibbing 2–3 seeds for each hole at a depth of 2–3 cm for each ridge with 15 cm plant spacing. Furthermore, four irrigations totaling 330 mm were applied throughout the crop, including the pre-sowing irrigation suggested as ideal for quinoa growing under Pakistani conditions (Akram et al., 2021).

#### Selection

A selective approach was employed to assess phenotypic diversity and ensure homogeneity, focusing on fewer plants while maximizing the uprooting of individuals. The panicles of these selected plants were bagged within 100 μm mesh pollination bags before the flowering stage [at BBCH 60 as described by Sosa-Zuniga et al. (2017)] to avoid heterogeneous seeds. After the flowering stage [at BBCH 70, as described by Sosa-Zuniga et al. (2017)], bags were removed to allow the panicles to expand and grow. After the removal of the bags, each plant was tagged based on phenotypic diversity to ensure the harvesting of panicles for pure seeds.

#### Data collection

Eighteen phenotypic traits, including qualitative and quantitative traits, were recorded to assess the variability among quinoa accessions. Phenotypic descriptors (Stanschewski et al., 2021) were used for data recording of qualitative traits. The phenotypic traits included 8 qualitative traits (panicle color, PC; panicle shape, PS; panicle density, PD; panicle leafiness, Pl; stem color, SC; leaf shape, LS; leaf margins, LM; and leaf granule color, LGC and 10 quantitative traits (number of branches, NOB; number of panicles, NOP; plant height, PH; stem diameter, SD; panicle length, PL; days to maturity, DM; days to flowering, DF; 1,000 seed weight, SW; yield per plant, YPP; and productivity, P). The data for days to flowering were collected when approximately half of the plants were at the flowering stage (Tabatabaei et al., 2022). Moreover, days to maturity were recorded when 90% of the plants in a plot attained maturity (El-Harty et al., 2021).

#### Data analysis

Phenotypic data of qualitative variables were analyzed using PAST software (Version 3.16) for dendrogram and principal component analysis. For quantitative data, hierarchical clustering heatmaps were generated in TBtool software. Spearman correlation analysis was performed in the R package “corrplot” in the R 4.1.3 program. Principal component analysis, eigenvalues, scree plots, and biplot analysis were also performed in the R 4.1.3 program using “factoextra,” “factorMineR,” and “GGbiplot,” respectively. These PCAs were generated from a correlation matrix created in R 4.1.3.

Furthermore, the “Nbclust” package was employed to calculate the optimum number of clusters for individual and variable biplot analysis. The elbow method from the “Nbclust” package was utilized to identify the optimum number of clusters. The data were analyzed to calculate the genetic distance matrix using the Euclidean distance method for creating a cluster dendrogram using the “ggplot2,” “factoextra,” and “ggsci” packages in R 4.1.3.

### Genotyping

#### DNA isolation

Fresh and young quinoa leaves (at the juvenile stage) were used for genomic DNA isolation. Fresh leaves were washed with distilled water and ground using a mortar and pestle. The ground material was subjected to DNA isolation using the GeneJET Genomic DNA Purification Kit following the manufacturer’s protocol. The purified DNA was stored at −20 °C for downstream analysis. The purified DNA was run on a 0.8% gel, and its quality and integrity were assessed through a gel documentation system (GDS). The DNA concentration was measured by UV visible NANODROP (8000 Spectrophotometer, Thermo Scientific), and a working dilution of 50 ng/μl was made.

#### PCR analysis

For genotypic analysis, polymerase chain reaction (PCR) was performed on a 96-well thermal cycler (peqSTAR) using 20 ISSR markers (Supplementary Table 2). The PCR mixture of 30 μl included template DNA (50 ng/μl), dNTPs, primer pair (each of 10 μM), MgCl_2_, *Taq* Polymerase, *Taq* buffer, and nuclease-free water to make the final volume. The PCR thermal profile consisted of an initial denaturation temperature at 94°C for 5 min, followed by 40 cycles of denaturation at 94°C for 1 min, annealing at 51°C for 1 min, and extension at 72°C for 2 min, and a final extension at 72°C for 15 min. PCR products were resolved on a 2.5% (w/v) high-resolution agarose gel (biotech grade, ACTGene) and visualized on a Gel Documentation system (Bio-Rad, USA).

#### Data analysis

Unambiguous DNA bands were counted and scored in the form of a binary matrix in Excel (MS toolkit) as “1” (presence) and “0” (absence). The collected data were aligned and analyzed using PAST software (Version 3.16). PowerMarker (Version 3.25) software computed major allele frequency, genetic diversity, heterozygosity, and polymorphic information content (PIC). Principal coordinate analysis (PCoA) was performed using DARwin6 software. The similarity matrix was generated in Pop Gen32 (Version 1.32) based on Nei’s original measure. STRUCTURE software (Version 2.3.4) was used to assess the pattern of genetic structure in a population, and STRUCTURE harvester software was used to assess the exact number of subpopulations (K). The number of subpopulations (K) was set from 2 to 10, with 10 replications for each run. The program was run with parameters of 10,000 burn-in periods followed by 10,000 iterations. Furthermore, STRUCTURE harvester software was used to assess subpopulations’ exact number (K) (Earl and VonHoldt, 2012).

#### Linkage disequilibrium

The linkage disequilibrium (LD) between all pairs of ISSR markers was calculated with 10,000 permutations using correlation coefficient (*r*^2^) in TASSEL software (Version 5.2.90) (Bradbury et al., 2007). The significance (*p*-value) level of *r*^2^ was estimated using Fisher’s exact test. The marker pairs in the same linkage group were considered linked markers; otherwise, they were considered unlinked markers. The LD level for linked and unlinked markers was also calculated using TASSEL software. Allelic pairs with *p* ≥ 0.0001 were considered to be in significant LD. The LD decay was estimated using the “LOESS” function in the R 4.1.3 program, and a trend line illustrating the LD decay was plotted using the *r*^2^ values against the genetic distance of loci pairs (bp). The crucial threshold below which the LD might be regarded as being caused by physical linkage was calculated, corresponding to the 95th percentile of the distribution of square root (*r*^2^). The LD decay value for locus pairs was calculated where the LD curve and the *r*^2^ threshold intersected.

#### Association mapping

The phenotypic and ISSR marker data were combined for association mapping. The association study was conducted using a general linear model (GLM) with a Q-matrix and mixed linear modes (MLM) with a population’s genetic structure (Q) and kinship (K) matrix following the permutation tests of 10,000 (Bradbury et al., 2007). A kinship matrix (K) was generated from 20 ISSR markers and 18 phenotypic variables. The Structure Harvester program calculated the population’s genetic structure (Q) matrix. Subsequently, trait-associated markers were subjected to filtration to evaluate their *r*^2^ values for GLM with significance levels *p* ≤ 0.01 and MLM with significance levels *p* ≤ 0.01 and *p* ≤ 0.05, eliminating markers with lesser statistical significance.

## Results

### Germplasm collection and characterization for screening

In genetic studies, heterogeneity poses considerable challenges because the phenotype must be correlated with the genotypic information. Hence, highly heterogeneous genotypes are not suitable for genetic studies. Quinoa is predominantly self-pollinating and has varying rates of natural hybridization of 10%–17%, which are likely to be greater at lower plant spacing and depend on the coincidence of flowering with the windiness of the site or the presence of other pollen vectors. The genetic diversity of quinoa is wide owing to less-intensive breeding events (and thus a relative paucity of population bottlenecks), and several quinoa accessions are landraces that produce a heterogeneous phenotype (Stanschewski et al., 2021). For this reason, quinoa germplasm was screened for homogeneity by morpho-agronomic and genomic characterization.

#### Phenotyping

As heterogeneity is a strong bottleneck for genetic study, each accession was sown in isolated plots in the field to ensure the homogeneity of collected seed samples. Among 43 accessions, populations of 18 accessions (CHEN 425, D-12175, CHEN 391, D-12220, CHEN-297, CHEN-128, CHEN-470, CHEN-33, CHEN-71, Ames-13760, PUC-mix-red, Javi, PI-614927, RU-5, D-12014, PI-614885, PI-614882, and Ames-13731) in 18 isolated plots were grown in the field (Supplementary Table 3). Heterogeneity was scored in each plot within the population of each accession. Plots were excluded from the analysis if >50% of the plants within a plot were segregating, i.e., they were observably different, and a plot was excluded when the main phenotype of the accession was not identifiable within the plot.

Fewer plants were selected to assess phenotypic diversity and ensure homogeneity and the maximum number were uprooted. Among the grown accessions, the populations of CHEN-425, D-12175, CHEN-391, D-12220, CHEN-128, CHEN-33, CHEN-470, CHEN-71, CHEN 297, Ames-13760, PUC-mix-red, Javi, PI-614927, and RU-5 were observed to be homogenous within their respective plots. Based on scored percent homogeneity, plants for each accession were selected, tagged, and bagged before BBCH 60 (Supplementary Table 3); however, the remaining plants were uprooted. Moreover, four plots for accessions D-12014, PI-614885, PI-614882, and Ames-13731 showed >50% heterogeneity and were excluded from the experiment.

**FIGURE 1:**
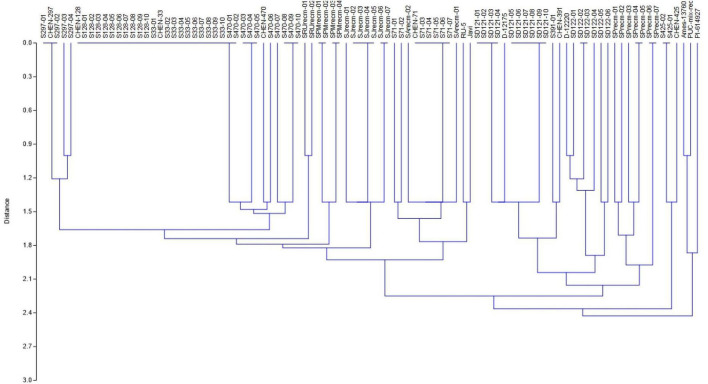
Unweighted pair group method arithmetic mean average (UPGMA) dendrogram of quinoa accessions based on phenotypic data under the standardized Euclidean distance coefficients using PAST software (Version 3.16).

For phenotyping, cluster analysis was performed on phenotypic data collected from the field-grown accessions and the literature documented for these accessions. The cluster analysis showed that phenotypic data of the tagged plants from the plots of CHEN-425, D-12175, CHEN-391, D-12220, CHEN-128, CHEN-33, CHEN-470, CHEN 297, and CHEN-71 were congruent with the data documented in the literature. However, the phenotypic data for accessions PI-614927, RU-5, PUC-mix-red, JAVI, and Ames-13760 deciphered incongruence with their documented phenotypic data. In cluster analysis, these accessions showed phenotypic divergence from the plants in their respective plots. It might be due to outcrossing and natural hybridization that led to recombinant line development. Therefore, these plants were labeled distinctly and used individually in the genetic study (Figure 1).

### Phenotypic characterization based on qualitative traits

#### Distribution frequency and diversity index

The phenotypic traits displayed significant and wide-ranging variations among the 72 quinoa plants. These variations could be important for developing new cultivars with distinctive morphological and agronomic characteristics. The PC demonstrated high variations among mature quinoa plants. Notably, most plants had green panicles (58%) with a high distribution frequency. However, plants characterized by dark-colored or beige/white panicles had the lowest distribution frequency (1%). For PS, 72% of the plants were intermediate, while only 4% were amarantiform. Pl is categorized as less (75%) with a high distribution frequency; conversely, only 6% of the plants had more Pl with the lowest distribution frequency. For PD, 69% of plants were intermediate, 28% compact, and 4% lax. SC showed green as the most frequent color (67%), while the lowest frequency distribution (14%) was observed for yellow stems. Leaf shape was divided into two major categories: 60% of the plants were rhomboidal, and 40% were triangular, which could be polymorphic for the same plant. The most common leaf edges were dentate (57%), with the highest distribution frequency, and only 11% of the plants had entire leaf edges. For LGC, white was the most frequent color in 81% of the plants, while only 1% had purple leaf granules. The Shannon diversity indices (SDI) for eight qualitative traits varied (*H* > 0.5) for all study descriptors, ranging from 1.706 for white PC to 3.574 for LS.

Qualitative traits and their respective descriptor states, frequency distribution (%), and Shannon diversity index (SDI) among different quinoa plants are summarized in Table 1.

**TABLE 1:** Absolute and relative frequency (%) distributions and Shannon diversity index (H′) of eight qualitative trait descriptors of quinoa plants.

Qualitative traits/botanical descriptors	Botanical descriptors states	Frequency %		H′
		**Absolute**	**Relative**	
Panicle color	Green	42	58	1.706
	Pink/purple/red	13	18	
	Yellow/orange	10	14	
	Green with purple	5	7	
	Dark colored	1	1	
	Beige/white	1	1	
Panicle shape	Glomerulate	17	24	2.703
	Intermediate	52	72	
	Amarantiform	3	4	
Panicle leafiness	More	4	6	2.581
	Median	14	19	
	Less	54	75	
Panicle density	Compact	20	28	2.533
	Intermediate	50	69	
	Lax	2	3	
Stem color	Green	48	67	3.033
	Yellow	10	14	
	Red	14	19	
Leaf shape	Rhomboidal	43	60	3.574
	Triangular	29	40	
Leaf margin	Dentate	41	57	3.031
	Serrate	23	32	
	Entire	8	11	
Leaf granule color	White	58	81	2.202
	White-red	13	18	
	Purple	1	1	

#### Principal component analysis

**FIGURE 2:**
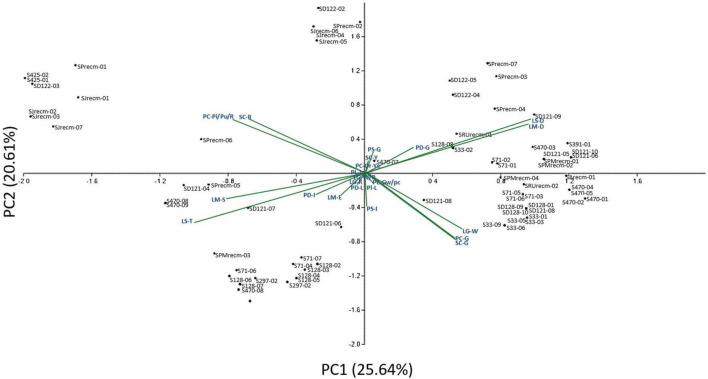
Two-dimensional ordination of the qualitative traits in quinoa plants. In component 1 (PC1), qualitative traits contributed more (25.64%) to the variation among genotypes, while qualitative traits in component 2 (PC2) contributed comparatively less (20.61%) of the total variance.

Principal component analysis (PCA) was performed to comprehend how the qualitative traits contribute to the variation among various genotypes. Principal component analysis explained 25.64% of the total variation by PC1 and 20.61% by PC2, accounting for 46.25% of the variance (Figure 2). These axes, which include PC, PS, Pl, PD, LS, LM, LGC, and SC, display significant differentiating qualitative traits. The qualitative traits that contributed more to the variation in PC1 include PC-G (green), Pl-L (less), SC-G (green), PS-In (intermediate), LS-R (rhomboidal), LM-D (dentate), and PD-C (compact) in PC1, while PC-Pi/Pu/R (pink/purple/red), SC-R (red), LM-S (serrate), LS-T (triangular), and PD-I (intermediate) primarily contributed to the variation in PC2. Furthermore, panicle color was highly correlated with stem color among various plants.

### Phenotypic characterization based on quantitative traits

#### Distribution frequency and descriptive statistics

**FIGURE 3:**
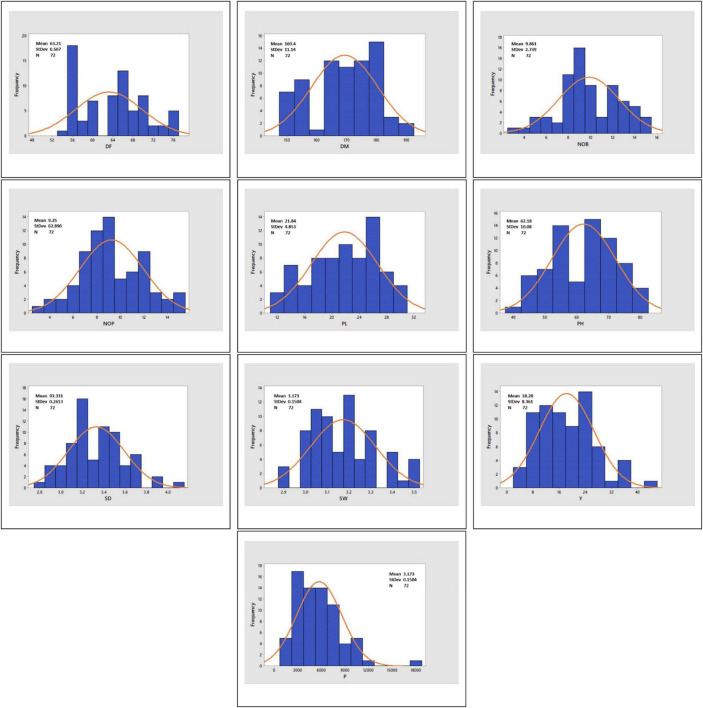
Histograms showing the frequency distribution curve for DF, days to flowering; DM, days to maturity; NOB, number of branches; NOP, number of panicles; PL, panicle length; PH, plant height; SD, stem diameter; SW, seed weight; Y, yield; and P, productivity.

Histograms with continuous distribution for all the quantitative variables were generated, displaying a wide range of phenotypic variation across the quinoa accessions (Figure 3). Descriptive statistics, including minimum and maximum range, mean, mean standard error, standard deviation, variance, coefficient of variation, data distribution pattern (skewness and kurtosis), and diversity indices (SDI and E_*H*_), are given in Table 2. For the coefficient of variation, a high level of phenotypic variability was observed in P (49.74%) and Y (45.71%), followed by moderate levels of variability in NOP (29.07%), NOB (27.77%) and PL (22.23%) and low levels of variability in PH (16.21%), DF (10.39%), SD (7.84%), DM (6.58%), and SW (4.74%). Most of the quantitative variables observed a low level of phenotypic variation. For the distribution pattern, skewness and kurtosis coefficients were formulated to construe the nature of the distribution for quantitative variables. The skewness value ranged from 0.5 to −0.5 for all the quantitative variables except for P (1.20). Likewise, all quantitative traits exhibited kurtosis <0, except for P (2.76). The Shannon diversity indices (SDI) for 10 quantitative traits varied (*H* > 0.5) for all study descriptors, ranging from 4.16 for production to 4.28 for SW and DM. Moreover, the evenness (E_*H*_) results likewise showed a high variation index value for all quantitative traits and ranged from 0.89 to 1.00. The highest E_*H*_ value was recorded at 1.00 for SD, SW, and DM, whereas the lowest E_*H*_ was 0.89 for P.

**TABLE 2:** Descriptive statistics and diversity indices (Shannon diversity index and evenness) for quantitative traits among 72 quinoa plants.

Quantitative traits/botanical descriptors	Min	Max	Mean	SE mean	SD	Var	CV	Skewness	Kurtosis	H′	E_H_
NOP	3.00	15.0	9.25	0.32	2.689	7.23	29.07	0.16	0.24	4.23	0.95
NOB	3.00	15.0	9.86	0.32	2.739	7.50	27.77	−0.09	−0.31	4.24	0.96
DM	148	189.0	169.3	1.31	11.14	124.1	6.58	−0.42	−0.83	4.28	1.00
DF	54.0	76.0	63.2	0.77	6.567	43.13	10.39	0.21	−1.03	4.27	0.99
PH	39.5	81.6	62.18	1.19	10.08	101.6	16.21	−0.21	−0.73	4.26	0.98
PL	12.4	30.6	21.8	0.57	4.853	23.6	22.23	−0.30	−0.85	4.25	0.97
SW	2.90	3.52	3.17	0.02	0.1504	0.02	4.74	0.48	−0.29	4.28	1.00
SD	2.80	4.10	3.33	0.03	0.2613	0.07	7.84	0.42	0.13	4.27	1.00
Y	5.21	42.72	18.29	0.99	8.361	69.91	45.71	0.59	−0.00	4.17	0.90
P	1,532	17,375	5,734	336	2,852	8,136,148	49.74	1.20	2.76	4.16	0.89

SE, standard error; Var, variance; CV, coefficient of variation; E_H_, evenness.

#### Spearman correlation analysis

**FIGURE 4:**
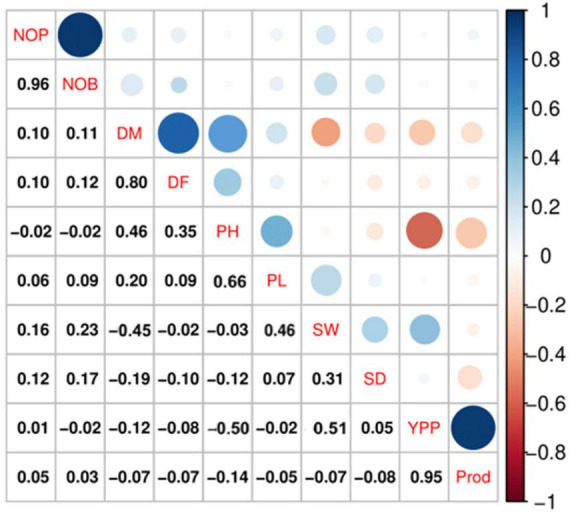
Spearman correlation analysis at a significance level of *p* ≤ 0.001 indicated significant correlations among the qualitative traits in quinoa plants. According to the color scale, a positive correlation is indicated by blue, and a negative relation is indicated by red. The circle size and intensity are relatively proportional to the correlation level.

Spearman correlation analysis explains the significant relationship among the quantitative traits. The Spearman correlation coefficient (*r*) at the significance level (*p* ≤ 0.001) for quantitative traits is represented in Figure 4, which shows the highest positive significant correlations between NOB and NOP (*r* = 0.96, *p* ≤ 0.001), P and YPP (*r* = 0.95, *p* ≤ 0.001), followed by DM and DF (*r* = 0.80, *p* ≤ 0.001). There were also significant positive correlations between DM and PH (*r* = 0.46, *p* ≤ 0.001) and between PH and PL (*r* = 0.66, *p* ≤ 0.001). However, a negative significant correlation was observed between PH and YPP (*r* = −0.50), followed by DM and SW (*r* = −0.45).

#### Principle component analysis

**FIGURE 5:**
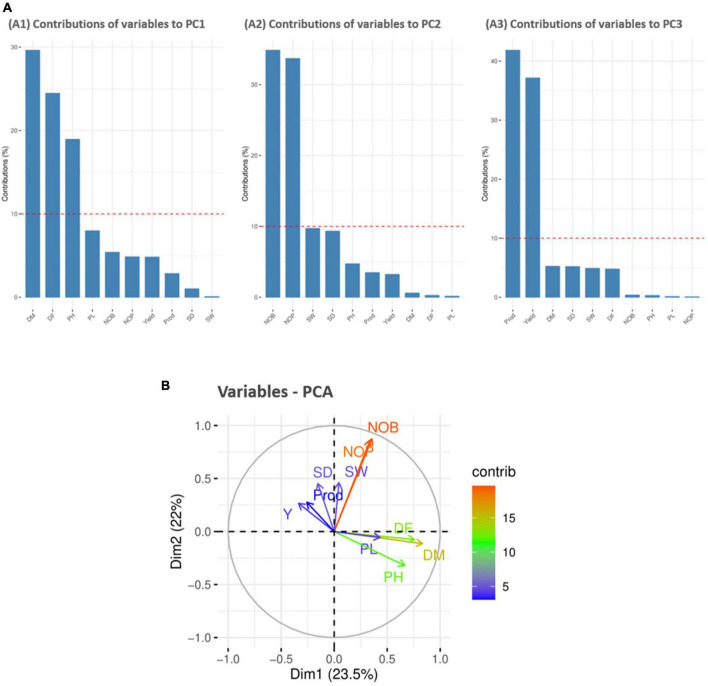
Contribution of variables to principal components (%). Red dashed lines across bar plots are the reference lines, and the variable bars above the reference lines are considered important in contributing to the respected PCs. DM, DF, and PH contributed more to the variation in PC1 **(A1)**, NOB and NOP contributed in PC2 **(A2)**, and P and Y contributed in PC3 **(A3)**. **(B)** Principal component analysis (PCA) score plots of quantitative variables in first two principal components (PC1 and PC2).

For variable principal component analysis, the results showed that DM, DF, and PH primarily contributed to PC1 (Figure 5A1); however, NOB and NOP showed major contributions to the overall phenotypic diversity in PC2 (Figure 5A2). Furthermore, P and Y were the distinctly contributing variables in PC3 (Figure 5A3). Furthermore, the score and loading plots of the PCA plot based on the quantitative data are presented in Figure 5B. In variable PCA, the first two dimensions (PC1 – 23.5% and PC2 – 22%) accounted for the largest variation in the overall variance. The PCA was represented as a correlation circle where quantitative variables were graphically represented by normalized vectors, depicting the correlation and contribution of variables (Figure 5B). In PC1, DF, DM, PH, PL, NOB, NOP, and SW contributed positively, whereas Y, P, and SD contributed to positive variance in PC2. In the correlation circle, each variable’s significance was strongly correlated with the length and direction of the vectors. Two variables (NOB and NOP) showed higher magnitudes with longer vector lengths (more variance), followed by DF, DM, and PH. Conversely, SW, SD, P, Y, and PL showed lower magnitudes with shorter vector lengths (less variance). Depending on the vector angles, two variables (NOP and NOB) had closer vector angles that showed a strong positive correlation. Furthermore, Y, SD, and SW also showed strong correlations with each other but a negative association with PL.

**FIGURE 6:**
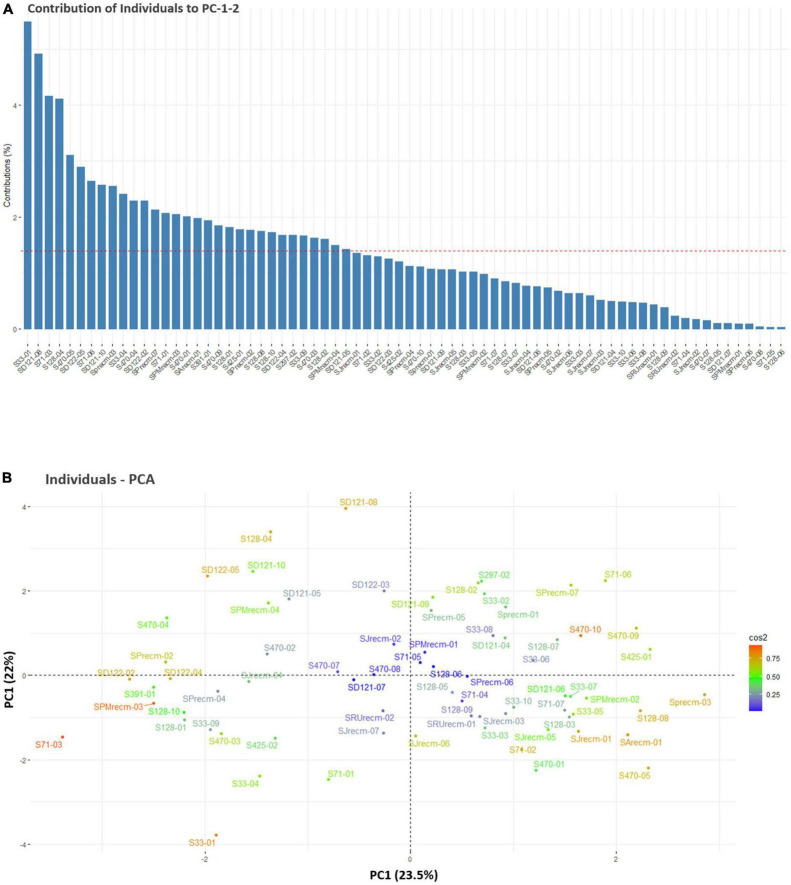
Contribution of individual plants to PC1 and PC2 (%). **(A)** Red dashed lines across bar plots are the reference lines, and individuals bars above the reference lines are considered important in contributing to PC1 and PC2. **(B)** Principal component analysis (PCA) score plots of individual’s distribution based on the first two PCs.

For individual PCA, S33-01, SD121-08, S71-03, S128-04, S470-05, SD122-05, S71-06, SD121-10, SPrecm-03, S33-04, S470-04, SD122-02, SPrecm-07, S71-01, SPMrecm-03, S470-01, SArecm-01, S291-01, S470-09, S128-01, S425-01, SPrecm-02, S128-08, S128-10, SD122-04, S297-02, S33-09, S470-03, S128-02, SPMrecm-04, and SD121-05 plants primarily contributed to PC1 and PC2 (Figure 6A). The score and loading plots of the PCA plot based on the quantitative data of quinoa plants are presented in Figure 6B. In individual PCA score plot, the first two dimensions (PC1 – 23.5% and PC2 – 22%) accounted for the largest variation in the overall variance. The results showed that all plants were effectively separated in all quadrants based on the first two principal components that showed phenotypic variability among them (Figure 6B).

**FIGURE 7:**
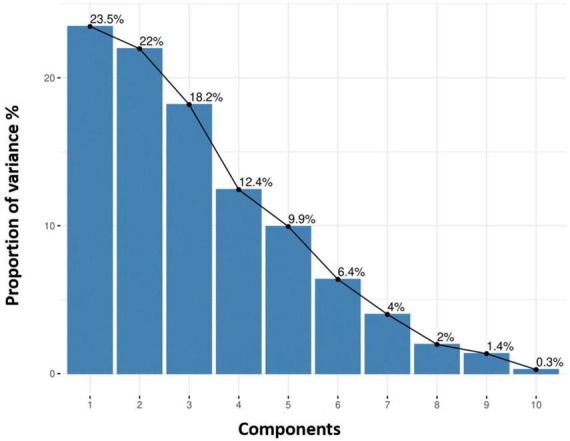
Each principal component explains the proportion of variance (%). The cumulative variation of PC1–PC2 is shown, where the black line describes the variance (%) in phenotypic diversity of quinoa accessions by each component.

The comparison of eigenvalues and proportion of variances for 10 principle components among quinoa accessions is given in Supplementary Table 4. A scree plot for quantitative traits was created based on the 10 PCs. The results showed that the first four PCs based on the quantitative data (eigenvalue >1) accounted for 76.1% of the total variance among all the accessions (Figure 7). Individually, PC1, PC2, PC3, PC4, PC5, PC6, PC7, PC8, PC9, and PC10 represented 23.5%, 22%, 18.2%, 12.4%, 9.9%, 6.4%, 4%, 2%, 1.4%, and 0.3% of the total variance, respectively.

#### PCA biplot analysis

**FIGURE 8:**
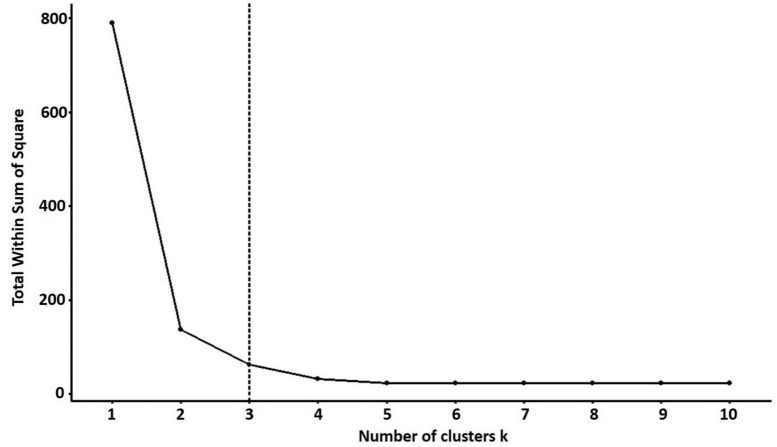
The optimal number of clusters is identified using the elbow method for quantitative traits. The optimal “*k*” is selected when the marginal gain noticeably slows down, creating an angle in the graph. The optimal *k* value is *k* = 3.

For PCA biplot analysis, the elbow method was used for the cluster optimization to delineate the level at which most phenotypic data is retained with well-explained phenotypic variability among quinoa accessions. The results showed that the optimal number of clusters was *k* = 3 to perform the cluster analysis, which shows the variations based on quantitative traits within the clusters (Figure 8).

**FIGURE 9:**
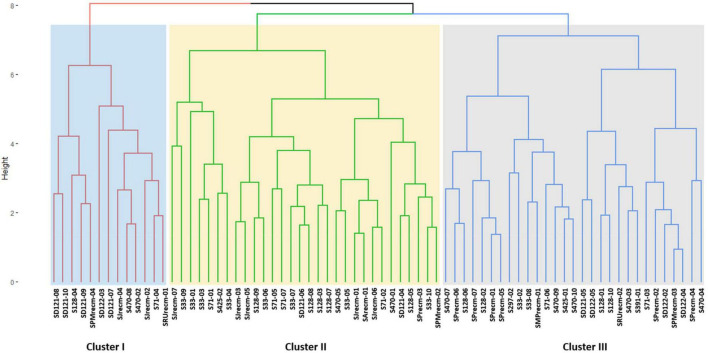
Clustering pattern of the 72 plants based on quantitative traits using Euclidean distance and ward clustering at a dissimilarity coefficient of 0.409.

Cluster analysis was performed for quantitative data that grouped all the quinoa plants into three distinct clusters (cluster I, cluster II, and cluster III) (Figure 9). The clustering tree (cluster dendrogram) showed that similar plants tend to group in the same cluster. The highest number of plants was grouped in cluster II, which comprised 30 plants, followed by cluster III with 29 plants, although cluster I showed the least number of plants (Figure 9).

**FIGURE 10:**
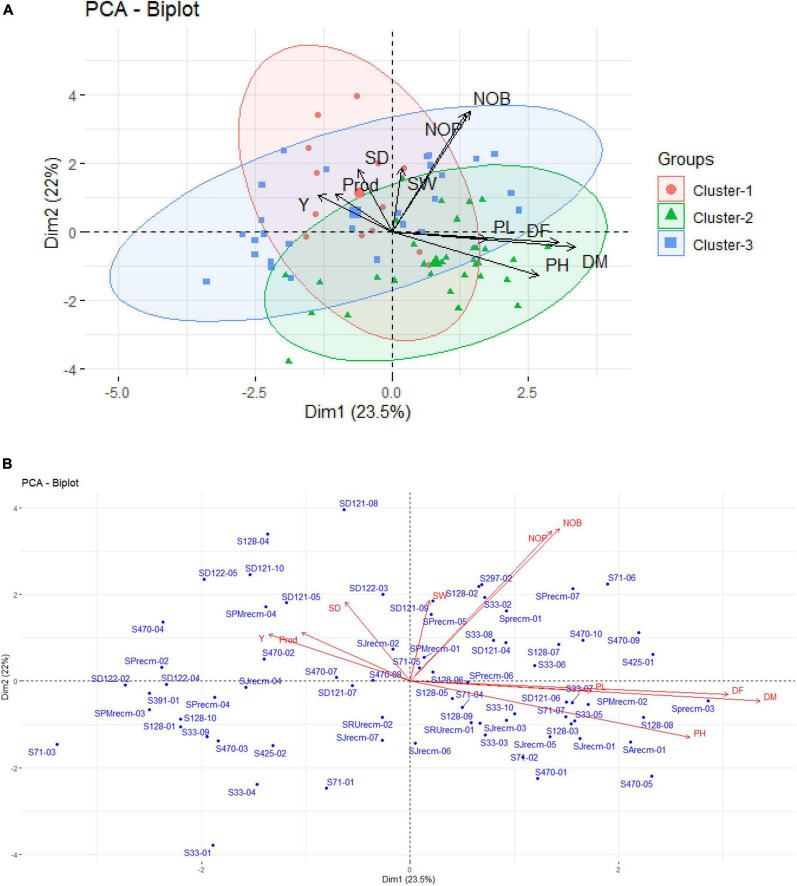
**(A)** Principal component analysis ellipse biplot demonstrating the clustering pattern of quinoa plants categorized by quantitative traits with different colors and symbols assigned to each cluster. **(B)** PCA biplot representing the quinoa plants from each accession with a particular trait.

In the PCA biplot, PC1 and PC2 accounted for 23.5% and 22% of the total phenotypic variance, respectively. The results showed that the plants from each accession were disseminated across all quadrants of the PCA ellipse plot and presented diverse clustering in the PCA biplot (Figure 10A). However, plants correlated with a particular varietal cluster primarily employed certain quadrants based on quantitative variables. From 72 quinoa plants, 13 plants (S71-04, SD121-07, SD121-08, SD121-09, SD121-10, SD122-03, S128-04, S128-04, S470-02, S470-08, SJrecm-02, SJrecm-04, SRUrecm-01, and SPMrecm-04) were closely grouped in cluster I and predominantly correlated with Y, SW, P, and SD, which showed that these plants might be crucial for generating high-yielding quinoa varieties (Figure 10B). However, most of the plants (S71-02, S71-04, S71-07, S128-03, S128-05, S128-08, S128-09, S33-03, S33-05, S33-07, S33-10, S33-07, SD121-06, SRUrecm-01, SJrecm-01, SJrecm-03, SJrecm-05, SJrecm-06, SPrecm-03, SPMrecm-02, SArecm-01, S470-01, and S470-05) in cluster II were correlated with DF, DM, PL, and PH, demonstrating that these plants were either early maturing or late maturing along with longer or shorter plant height and panicle length. In cluster III, 17 plants (S297-02, S71-06, SPrecm-01, SPrecm-05, SPrecm-07, SMPrecm-01, S33-02, S33-06, S33-08, S470-09, S470-10, S128-02, S128-06, S128-07, S128-08, SD121-04, and S425-01) were highly correlated with NOP and NOB than Y, SW, P, and SD.

#### Heatmap and hierarchical cluster analysis

**FIGURE 11:**
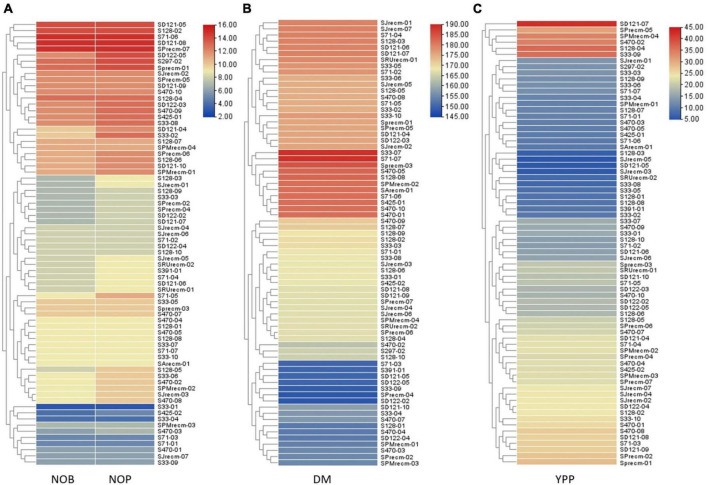
Heatmap presenting the clustering pattern of 72 quinoa plants and their correlation with traits analyzed **(A)** number of branches (NOB) and number of panicles (NOP), **(B)** days to maturity (DM), and **(C)** yield per plant (YPP), where different colors and their intensity on right panel represent the range of quantitative traits.

Heatmap cluster analysis was performed for quinoa plants and their quantitative traits, including NOB and NOP, DM, and YPP. The heatmap along the dendrogram showed the grouping of quinoa plants based on particular quantitative traits (Figures 11A–C). The clustering heatmaps (cluster dendrogram) categorized quinoa plants into two groups based on NOP and NOB (Figure 11A). Group 1 is the largest subdivided into two subgroups; subgroup 1 included 10 plants that varied in NOP and NOB from 2 to 6, while subgroup 2 included 36 plants that ranged from 6 to 10. Group 2 had 26 plants with the highest number of branches and panicles, ranging from 12 to 16. In group 2, two plants (S71-06 and SPrecm-07) had maximum number of branches and panicles (15). For DM, the clustering heatmap showed two major groups (Figure 11B). Group 1 was subdivided into two subgroups; subgroup 1 included 17 plants that matured early, ranging from 145 to 160 days, while, subgroup 2 included 23 plants that required 160–170 days to attain maturity. Group 2 was further divided into two subgroups. Subgroup 2 included 21 plants that reached the maturity stage in 170–185 days. Subgroup 1 included 11 late-maturing plants, requiring 185–190 days for maturity. Heatmap showed the most early maturing plants were SPrecm-04 and SD122-02 that attain maturity in only 148 days followed by S33-09 (149 days). A clustering heatmap for Y showed two major groups (Figure 11C). Group 1 was subdivided into two subgroups; subgroup 1 comprised 26 plants with the lowest Y value ranging from 5 to 15, and subgroup 2 included 40 plants ranging from 20 to 30. Group 2 included only six plants (SD121-07, S128-04, S33-09, S470-02, SPMrecm-04, and SPrecm-05) with the highest value for Y, ranging from average value reside within 30 to 45 g/1,000 seeds.

The phenotypic data and analysis showed strong correlation of panicle shape, color, density and leafiness (panicle architecture) with yield and productivity in quinoa. The accession SD-121-07 exhibited highest yield per plant (42.72 g; YPP) possessing green, glomerulate, compact panicle with less leaves followed by accessions S128-04 (36.88 g), S33-09 (36.24 g), S470-02 (34.44) with non-significant difference in yield having green, intermediate, intermediate panicles with less leaves. It showed that change in panicle shape and density from glomerulate shape, compact density panicle to intermediate shape, intermediate density panicles resulting in yield reduction. The accessions SJrecm-03 and SJrecm-05 produced less yield, 5.21 and 5.49 g, respectively. Both exhibited pink, intermediate shape, intermediate density panicles with less leaves. In addition to this, the results also inferred that the accessions with panicle shape amarantiform exhibited less yield (Supplementary Table 5).

### Genetic distance relatedness

**FIGURE 12:**
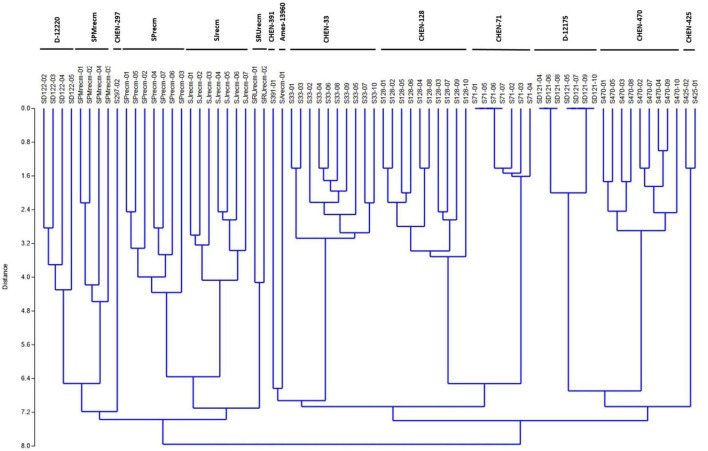
Unweighted pair group method arithmetic mean average (UPGMA) dendrogram of quinoa accessions based on scoring the DNA bands amplified in ISSR marker analysis using PAST software (Version 3.16).

Genetic relatedness was assessed using ISSR marker analysis in quinoa accessions (Figure 12). The cluster analysis was performed by consuming binary data generated by counting and scoring the DNA bands amplified in ISSR marker analysis using UPGMA (unweighted pair group method with arithmetic mean) based on the Jaccard similarity coefficients method to construct a dendrogram. In this study, the cluster analysis grouped the quinoa accessions into three major clusters (cluster I, cluster II, and cluster III). Cluster I comprised three accessions (CHEN-425, CHEN-470, and D-12175) originally from Peru. Cluster II comprised three accessions (CHEN-71, CHEN-128, and CHEN-33) from Bolivia, including one accession (CHEN-391) from Peru and one recombinant line (SArecm). Cluster III comprised six accessions, two from Peru (CHEN-297 and D-12220), and four were recombinant lines (SRUrecm, SJrecm, SPrecm, and SPMrecm) (Figure 12). Furthermore, the diversity parameters of the ISSR markers are given in Supplementary Table 6. A similarity matrix based on Nei’s (1972) genetic distance matrix of 72 quinoa plants is presented in Supplementary Table 7.

### Principal coordinate analysis

**FIGURE 13:**
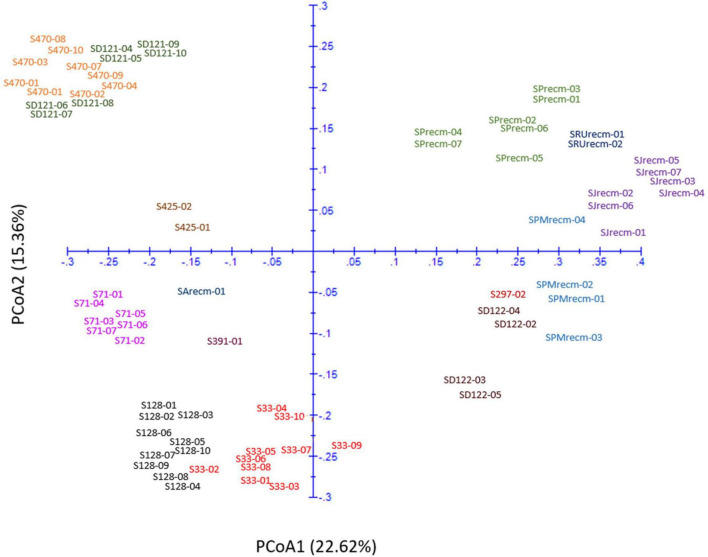
Two-dimensional plot of 72 quinoa plants from each accession based on genetic distance from ISSR data using principal coordinate analysis (PCoA).

Principal coordinate analysis was performed using the matrix calculated from ISSR marker data to compute the genetic diversity according to spatial patterns and genetic differentiation among quinoa accessions. The two-dimensional principal coordinate analysis showed that PCoA1 and PCoA2 accounted for 22.62% and 15.36% of the total genetic similarity variance, respectively (Figure 13). These results showed a strong concordance with the UPGMA cluster analysis. The PCoA plot presented a clustering of 14 quinoa accessions into three groups (Groups I, II, and III). Group I includes plants from CHEN-425, CHEN-470, and D-12175, representing a separate group originating mainly from Peru.

Furthermore, the plants from accessions CHEN-470 and D-12175 were genetically related, as they were more closely clustered than CHEN-425. The second group was mixed, originating from Bolivia, Peru, and the USA. In group II, plants from two accessions, CHEN-71 from Bolivia and CHEN-391 from Peru, and one recombinant line (SArecm) were genetically different, as they were distantly clustered. The plants from CHEN-128 and CHEN-33 were genetically related, as they were narrowly clustered and belonged to Bolivia and Peru origin, respectively. In group III, plants from two accessions, D-12220 and CHEN-297, were genetically more related, originating from Peru, than the recombinant lines (SPMrecm, SJrecm, SRUrecm, and SPrecm).

### Population structure

**FIGURE 14:**
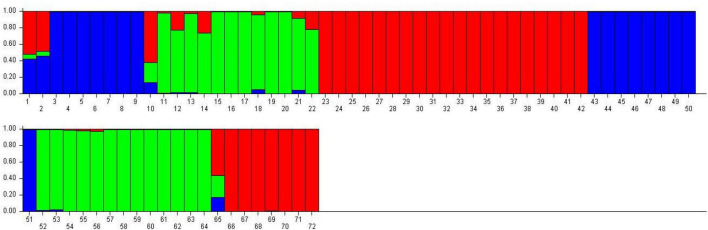
Classification of quinoa plants into three main populations using STRUCTURE 2.3.4 software. The color code indicates the distribution of the accessions to different populations. Numbers on the *y*-axis show the subgroup membership, and the *x*-axis shows the different accessions and population IDs in brackets.

Population structure analysis was performed using STRUCTURE software based on the Bayesian method to assess quinoa accessions’ genetic diversity and structure pattern (Figure 14). This clustering approach categorizes genotypes into distinct subpopulations. The Evanno method in the STRUCTURE program predicts the most likely number of clusters by analyzing the log probability of data for different *K* values and Δ*K* statistics (Evanno et al., 2005). In this study, the number of *K* was set from 2 to 10 with a burn-in period of 10,000 and an MCMC (Markov Chain Monte Carlo) run length of 10,000 for each run. The Evanno method estimated that the optimal “*K*” for representing the quinoa accessions was *K* = 3 (Supplementary Figure 1), thereby clustering these accessions into three main subpopulations and admixtures (Figure 14). The first subpopulation contained 27 plants (38%) grouped into subpopulation 1. In contrast, 25 (35%) were grouped into subpopulation 2. Furthermore, 16 plants (22%) were grouped into subpopulation 3, and only four plants (5%) were placed in admixture (Supplementary Table 8).

#### Linkage disequilibrium

**FIGURE 15:**
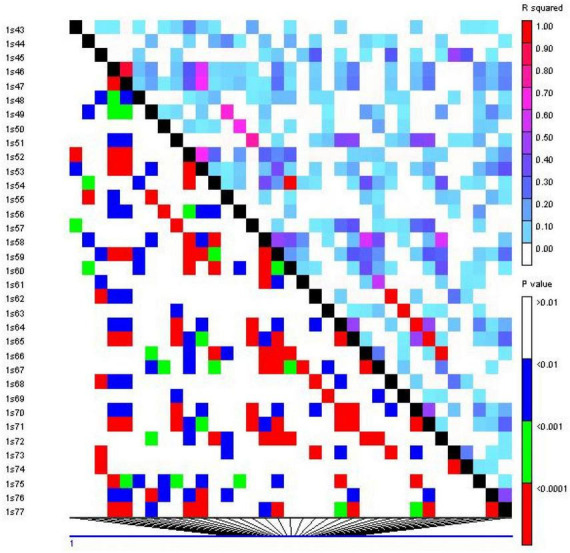
Linkage disequilibrium (LD) plot generated by 20 ISSR markers. The upper diagonal shows *r*^2^ among each pair of markers. The lower diagonal shows the significance levels (*p*-value) between each pair of markers.

**FIGURE 16:**
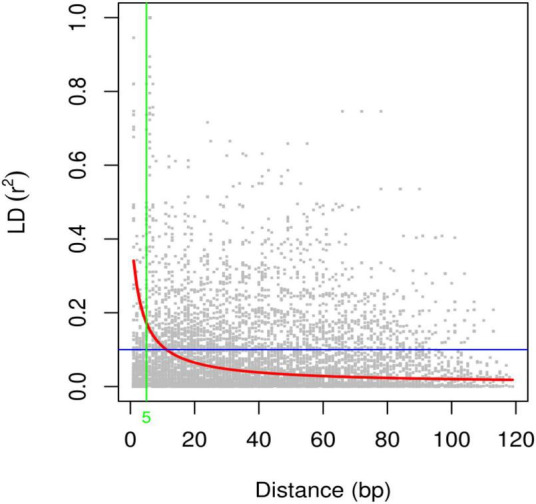
Scatter plot of the linkage disequilibrium (LD) decay with the critical *r*^2^ value and the genetic distance (bp) for ISSR markers. The red line shows the critical *r*^2^ value, i.e., 0.12.

Linkage disequilibrium (LD) analysis was performed for 20 ISSR markers using pairwise squared-allele frequency correlations (*r*^2^) to assess the LD level among different marker pairs (Figure 15). Based on significant LD (*p* ≤ 0.05, 0.0001), the marker combinations (%) were calculated from a total number of possible marker combinations. As a result, a total of 7,140 pairwise combinations were identified. Among these, 1,654 (23.16%) locus pairs showed significant LD at *p* ≤ 0.05, while 745 (10.43%) locus pairs showed strong LD at *p* ≤ 0.0001. Based on *r*^2^ at a significance level of *p* ≤ 0.0001, 4.1% and 2.7% of locus pairs showed significant LD with *r*^2^ < 0.2 and 0.3 > *r*^2^ > 0.2, respectively. Furthermore, 1.5% and 0.3% of locus pairs were in significant LD, with 0.5 > *r*^2^ > 0.3 and *r*^2^ > 0.5 (Supplementary Table 9). LD decay was assessed by plotting the *r*^2^ value against the genetic distance (bp) of locus pairs, and subsequently, a trend line was created to show the pattern of LD decay (Figure 16). LD relies on *r*^2^ values, and its critical threshold value (*r*^2^ = 0.12) was measured corresponding to the 95th percentile of the coefficient square (indicated by the red line in Figure 16), which showed that LD could be due to physical linkage. The LD decay value at the intersection of the LD curve and *r*^2^ threshold was attained at 5 bp for the whole genome.

### Marker-trait association analysis

For marker-trait association analysis, 20 highly polymorphic ISSR markers were employed in 72 quinoa plants to identify the significant markers for 18 phenotypic traits. Based on the GLM (general linear model), marker-trait associations (MTAs) were identified at a significance level of *p* ≤ 0.01 at 1% FDR (false discovery rate) after FDR correction (Supplementary Table 10). The results at *p* ≤ 0.01 showed that 10 ISSR markers were significantly associated with PC, while 13 markers were associated with SC. The highest number of ISSRs (16) was found to be associated with PS, followed by 15 ISSRs associated with Pl and 14 ISSRs linked with LGC. Furthermore, six and four ISSRs were associated with LS and PD. None of the ISSR markers was significantly associated with the stem LM trait. For quantitative traits, three markers were associated with PH, and two were linked to PL. A total of six ISSRs were identified for three yield-related traits, including two markers that were linked to productivity (P), three markers that were associated with seed weight (SW), and only one marker that was associated with DM. Moreover, no marker was found to be significantly associated with the NOB, NOP, DF, SD, or yield (Y) (Supplementary Table 10).

Based on MLM (mixed linear model), marker-trait associations (MTAs) were identified at *p* ≤ 0.01 and *p* ≤ 0.05 at 1% and 5% FDR, respectively, after FDR correction (Table 3). For qualitative traits, 12 ISSR markers were significantly associated with LGC at a significance level of *p* ≤ 0.05, while by increasing stringency, only 5 markers were significantly associated with LGC at *p* ≤ 0.01. Eight ISSR markers were associated with PC at *p* ≤ 0.05, and only four markers showed a significant association at *p* ≤ 0.01. Six markers showed a significant association for Pl at both significance levels (*p* ≤ 0.01 and *p* ≤ 0.05). Furthermore, four ISSRs were found to be associated with PS at *p* ≤ 0.05, and only two ISSRs were significantly associated at *p* ≤ 0.01. For PD, the lowest number of ISSRs showed a significant association at *p* ≤ 0.05; however, no ISSR marker was significant after increasing stringency at *p* ≤ 0.01. Furthermore, four ISSR markers were significantly linked to SC at *p* ≤ 0.05, and only one ISSR marker showed an association at *p* ≤ 0.01. Seven ISSR markers were found to be associated with LS at *p* ≤ 0.05, and one ISSR was significantly associated at *p* ≤ 0.01. For LM, two markers were associated at a significance level of *p* ≤ 0.05, and no ISSR showed a significant association at *p* ≤ 0.01 (Table 3). For quantitative traits, four ISSRs showed a significant association (*p* ≤ 0.05) with NOP, while two ISSRs were associated with NOB. Three ISSR markers were significantly associated with DM, and two ISSRs were associated with DF at *p* ≤ 0.05. Four markers were significantly associated (*p* ≤ 0.05) with PH and PL. Three markers were significantly associated with SW, whereas two were associated with trait P. The lowest number of markers (one ISSR) was found to be significantly associated with SD. None of the markers showed a significant association with trait Y. Moreover, by increasing stringency, none of the quantitative traits was significantly associated at *p* ≤ 0.01 (Table 3).

**TABLE 3:** Inter simple sequence repeat markers associated with qualitative and quantitative traits in quinoa plants at the significance level (*p* ≤ 0.05 and *p* ≤ 0.01) and phenotypic variance (*R*^2^) under MLM analysis.

Trait	*p* ≤ 0.05			*p* ≤ 0.01		
	**Marker**	***p*-Value**	***R* ^2^**	**Marker**	***p*-Value**	***R* ^2^**
Panicle color	ISSR1	0.01969	0.08498	^–^	^–^	^–^
	ISSR2	0.01969	0.08498	^–^	^–^	^–^
	ISSR3	0.03025	0.07293	^–^	^–^	^–^
	ISSR4	0.03025	0.07293	^–^	^–^	^–^
	ISSR5	0.00732	0.11395	ISSR5	0.00732	0.11395
	ISSR6	0.00732	0.11395	ISSR6	0.00732	0.11395
	ISSR17	0.00863	0.10902	ISSR17	0.00863	0.10902
	ISSR18	0.00863	0.10902	ISSR18	0.00863	0.10902
Stem color	ISSR7	0.03657	0.06625	^–^	^–^	^–^
	ISSR10	0.00119	0.16704	ISSR10	0.00119	0.16704
	ISSR17	0.01776	0.08601	^–^	^–^	^–^
	ISSR18	0.01776	0.08601	^–^	^–^	^–^
Panicle shape	ISSR15	0.00915	0.10697	ISSR15	0.00915	0.10697
	ISSR16	0.00915	0.10697	ISSR16	0.00915	0.10697
	ISSR17	0.03879	0.06593	^–^	^–^	^–^
	ISSR18	0.03879	0.06593	^–^	^–^	^–^
Panicle leafiness	ISSR8	0.00163	0.1215	ISSR8	0.00163	0.1215
	ISSR9	0.00319	0.10547	ISSR9	0.00319	0.10547
	ISSR15	0.00645	0.08906	ISSR15	0.00645	0.08906
	ISSR16	0.00645	0.08906	ISSR16	0.00645	0.08906
	ISSR17	0.00958	0.08013	ISSR17	0.00958	0.08013
	ISSR18	0.00958	0.08013	ISSR18	0.00958	0.08013
Panicle density	ISSR3	0.02722	0.07587	^–^	^–^	^–^
	ISSR4	0.02722	0.07587	^–^	^–^	^–^
Leaf shape	ISSR1	0.04534	0.06213	^–^	^–^	^–^
	ISSR2	0.04534	0.06213	^–^	^–^	^–^
	ISSR5	0.00145	0.16518	ISSR5	0.00145	0.16518
	ISSR7	0.03586	0.06851	^–^	^–^	^–^
	ISSR8	0.01465	0.09383	^–^	^–^	^–^
	ISSR11	0.02904	0.07434	^–^	^–^	^–^
	ISSR12	0.02905	0.07433	^–^	^–^	^–^
Leaf margin	ISSR19	0.02218	0.07741	^–^	^–^	^–^
	ISSR20	0.02218	0.07741	^–^	^–^	^–^
Leaf granule	ISSR3	0.0133	0.08027	^–^	^–^	^–^
	ISSR4	0.01332	0.08024	^–^	^–^	^–^
	ISSR5	0.0000259	0.25423	ISSR5	0.0000259	0.25423
	ISSR6	0.0000259	0.25421	ISSR6	0.0000259	0.25421
	ISSR7	0.01501	0.07735	^–^	^–^	^–^
	ISSR9	0.00231	0.12473	ISSR9	0.00231	0.12473
	ISSR11	0.0214	0.06889	^–^	^–^	^–^
	ISSR12	0.02142	0.06886	^–^	^–^	^–^
	ISSR17	0.0034	0.11461	ISSR17	0.0034	0.11461
	ISSR18	0.0034	0.11456	ISSR18	0.0034	0.11456
	ISSR19	0.0293	0.06155	^–^	^–^	^–^
	ISSR20	0.02932	0.06153	^–^	^–^	^–^
Number of panicles	ISSR11	0.03079	0.0705	^–^	^–^	^–^
	ISSR12	0.03079	0.0705	^–^	^–^	^–^
	ISSR17	0.04729	0.05913	^–^	^–^	^–^
	ISSR18	0.04729	0.05913	^–^	^–^	^–^
Number of branches	ISSR17	0.03358	0.06937	^–^	^–^	^–^
	ISSR18	0.03358	0.06937	^–^	^–^	^–^
Days to maturity	ISSR6	0.02116	0.08226	^–^	^–^	^–^
	ISSR13	0.04527	0.06143	^–^	^–^	^–^
	ISSR14	0.04527	0.06143	^–^	^–^	^–^
Days to flowering	ISSR13	0.01899	0.08313	^–^	^–^	^–^
	ISSR14	0.01899	0.08313	^–^	^–^	^–^
Plant height	ISSR6	0.04912	0.05413	^–^	^–^	^–^
	ISSR7	0.01426	0.08539	^–^	^–^	^–^
	ISSR19	0.01828	0.07891	^–^	^–^	^–^
	ISSR20	0.01828	0.07891	^–^	^–^	^–^
Panicle length	ISSR11	0.04203	0.06391	^–^	^–^	^–^
	ISSR12	0.04203	0.06391	^–^	^–^	^–^
	ISSR19	0.01027	0.10383	^–^	^–^	^–^
	ISSR20	0.01027	0.10383	^–^	^–^	^–^
Seed weight	ISSR11	0.03757	0.06594	^–^	^–^	^–^
	ISSR17	0.03311	0.06934	^–^	^–^	^–^
	ISSR18	0.03311	0.06934	^–^	^–^	^–^
Stem diameter	ISSR7	0.01934	0.08522	^–^	^–^	^–^
Productivity	ISSR15	0.02261	0.07864	^–^	^–^	^–^
	ISSR15	0.0133	0.09339	^–^	^–^	^–^
	ISSR16	0.02261	0.07864	^–^	^–^	^–^
	ISSR16	0.0133	0.09339	^–^	^–^	^–^

## Discussion

Phenotypic characterization of quinoa germplasm has been crucial for understanding their genetic diversity. It is imperative to uncover and comprehend the genetic variation at a broader level for efficiently assessing, preserving, and harnessing these germplasms (Manjarres-Hernández et al., 2021). In genetic studies, heterogeneity poses significant obstacles because the phenotypic and genotypic information must be associated. Hence, highly heterogeneous genotypes are unsuitable for genetic studies (Stanschewski et al., 2021). For this reason, quinoa germplasm must be screened for homogeneity by morpho-agronomic and genomic characterization. Globally, the prime objective of plant breeding programs is to breed for substantial yield, desired grain quality, and resilience to environmental stresses. Since the effectiveness of breeding programs widely hinges on the level of genetic variability, phenotypic characterization is considered the foremost step in delineating genetic resources (Carrillo-Perdomo et al., 2020). In the present study, 72 plants from 14 quinoa accessions were morpho-agronomically and genetically characterized using 18 phenotypic traits and 20 ISSR markers, respectively.

In the current study, phenotypic traits displayed significant and wide-ranging variations among the 72 quinoa plants. The PC demonstrated high variations among mature quinoa plants. Notably, most plants had green panicles (58%) and intermediate panicle shapes (72%). Pl was primarily categorized as lax (75%), while for panicle density, most of the plants (69%) were intermediate. Panicle phenotyping has been performed in previous research studies for quinoa accessions that also exhibited high variability in panicle shape, color, leafiness, and density (de Oliveira Vergara et al., 2020; El-Harty et al., 2021; Manjarres-Hernández et al., 2021; Craine et al., 2023). SC showed green as the most frequent color (67%). Leaf shape was divided into two major categories: 60% of the plants were rhomboidal, and 40% were triangular, which could be polymorphic for the same plant, with 57% having dentate leaf edges. For LGC, white was the most frequent color in 81% of the plants, while only 1% had purple leaf granules. These observations were validated by preceding studies that found various stem colors, leaf shapes and margins, and leaf granule colors (Bhargava and Ohri, 2016; El-Harty et al., 2021).

A PCA was performed to comprehend how the qualitative traits contribute to the variation among various genotypes. Principal component analysis explained 25.64% of the total variation by PC1 and 20.61% by PC2, accounting for 46.25% of the variance. The qualitative traits that primarily contributed to the variation in PC1 included PC-G, SC-G, LS-R, and LM-D in PC1, while PC-Pi/Pu/R, SC-R, LM-S, and LS-T contributed more to the variation in PC2. Furthermore, panicle color was highly correlated with stem color among various plants. Previously, El-Harty et al. (2021) observed variations among quinoa genotypes in PC1 (37.9%) associated with perigonium color, stem color, and leaf margins and in PC2 (27.3%) associated with leaf color and panicle color at flowering and maturity.

For quantitative traits, descriptive statistics demonstrated high genetic variation among quinoa accessions. The NOP and NOB ranged from 3 to 15 with coefficients of variation of 29.07% and 27.77%, respectively, verified by Coronado et al. (2020). The most variable descriptors were yield (g) with CV (45.71%), which ranged from 5.2 to 42.7, and productivity per plant (g) with CV (49.74%), which ranged from 1,532 to 17,375. In preceding studies, these variables were more discriminative for evaluating quinoa genotypes (Coronado et al., 2020). The CV values found for other quantitative traits were also verified by previously reported studies on quinoa characterization (Afiah et al., 2018; Coronado et al., 2020; Morillo-Coronado et al., 2022). The DM trait with less CV (6.58%) ranged from 148 to 189 days; however, this trait has been greatly distinctive in different geographical zones. In Saudi Arabia, quinoa plants require 98–177 days to mature, while they require 98–105 days in the USA (El-Harty et al., 2021), 107–158 days in Turkey (Mustafa and Temel, 2018), 109–163 days in India (Oustani et al., 2023), and the highest maturity duration from 108 to 182 days in Denmark (Sajjad et al., 2014). This extent of variability enables plants to hastily acclimatize to changing environmental patterns (Bazile et al., 2015). However, these factors serve as the foundation for genetic improvement initiatives since selection is impossible without variability because all individuals react similarly to the assessed conditions. Therefore, phenotypic variability in qualitative and quantitative traits enables the efficient selection of genetic material (Manjarres-Hernández et al., 2021).

The Spearman correlation analysis was employed for determining the degree of association between two variables, particularly in nonlinear relationships. The Spearman correlation coefficient (*r*) at the significance level (*p* ≤ 0.001) for quantitative traits showed the highest positive significant correlations between NOB and NOP, P and Y, followed by DM and DF. There were also significant positive correlations between DM and PH and between PH and PL. However, a high negative significant correlation was observed between PH and Y, followed by DM and SW. These results were consistent with previous studies that found a significant positive correlation between plant height and panicle length (Montes-Rojas et al., 2018) and a negative correlation between plant height and yield (Manjarres-Hernández et al., 2021). PCA is intended to better understand traits’ contribution to overall variance and characterize germplasm using qualitative traits (Nalajala et al., 2023). In the current study, PC1 and PC2 accounted for 23.5% and 22% of the total phenotypic variance, respectively. In PC1, DF, DM, PH, PL, NOB, NOP, and SW representatively contributed, whereas Y, P, and SD primarily contributed to the variance in PC2. These results showed strong congruency with a previous research study where plant height and number of panicles also contributed to PC1, while seed weight contributed to PC2 (Coronado et al., 2021).

In PCA biplot, the contribution and significance of each variable are shown by the vectors’ direction and length, which gives insight into variable contribution and its association with samples. For PCA biplot analysis, categorization through cluster analysis was performed to generate a stable grouping pattern based on similarities among quantitative traits (Ward, 1963). The cluster analysis was performed using the elbow method to estimate the optimum number of clusters (Sant’Anna et al., 2021). Furthermore, a PCA biplot was employed to assess the relationship between quinoa accessions and their agronomic traits. The results showed that the plants from each accession were distributed across all quadrants of the PCA ellipse plot, presenting diverse clustering (significant genetic diversity) in the PCA biplot. S71-04, SD121-07, SD121-08, SD121-09, SD121-10, SD122-03, S128-04, S128-04, S470-02, S470-08, SJrecm-02, SJrecm-04, SRUrecm-01, and SPMrecm-04 were closely grouped in cluster I and predominantly correlated with Y, SW, P, and SD, which showed that these plants might be crucial for generating high-yielding quinoa varieties. These results confirm previous findings for clustering many quinoa accessions by quantitative variables in a PCA biplot (Craine et al., 2023). For phenotyping quinoa plants with substantial agroeconomic traits, the quantitative variables that should be taken into consideration are plant height, panicle length, seed weight, stem diameter, and yield, while qualitative variables include panicle density (Manjarres-Hernández et al., 2021).

Phenotypic characterization of quinoa germplasm has been crucial for understanding their genetic diversity. It is imperative to uncover and comprehend the genetic variation at a broader level for efficiently assessing, preserving, and harnessing these germplasms (Haq et al., 2021; Manjarres-Hernández et al., 2021). Globally, the prime objective of plant breeding programs is to breed for substantial yield, desired grain quality, and resilience to environmental stresses. Since the effectiveness of breeding programs widely hinges on the level of genetic variability, phenotypic characterization is considered the foremost step in delineating genetic resources (Carrillo-Perdomo et al., 2020). In the present study, 72 quinoa accessions were genetically characterized using 18 phenotypic traits and 20 ISSR markers, their association with distinct agronomic traits.

Plant populations frequently exhibit structured populations due to intentional selection, non-random mating, and geographical segregation. Genetic markers may, therefore, appear to be falsely linked to particular traits. It may happen if researchers do not adequately consider or adjust for the impact of population structure in their findings (Mohamed et al., 2020). Therefore, population structure must be considered to prevent reporting false positive associations between genetic markers and traits. As a result, assessing the population structure is essential in association mapping analysis (Alemu et al., 2020).

This study employed ISSR markers to assess the genetic relatedness and population structure of 72 quinoa accessions. The cluster analysis grouped the quinoa accessions into three major clusters (cluster I, II, and III). Cluster I comprised three accessions (CHEN-425, CHEN-470, and D-12175) originally from Peru. Cluster II comprised three accessions (CHEN-71, CHEN-128, and CHEN-33) from Bolivia, including one accession (CHEN-391) from Peru and one recombinant line (SArecm-01). Cluster III comprised six accessions, two from Peru (CHEN-297 and D-12220), and four were recombinant lines (SRUrecm, SJrecm, SPrecm, and SPMrecm). El-Harty et al. (2021) reported that ecological diversification enhanced quinoa diversity and that UPGMA cluster analysis grouped quinoa accessions into three major clusters, where the genotypes in cluster I were from Peru, those in cluster II were from Egypt, Bolivia, and the USA. Those in cluster III were from Bolivia and Ecuador (El-Harty et al., 2021). However, the distribution of some genotypes did not strictly align as per geographical origins, mainly because these genotypes were adaptable to a wide range of agroecological circumstances and the probability of seed exchange. As quinoa is allotetraploid; hence, SSR markers may possibly produce four or more amplicons that becomes genotyping data more challenging to understand and record (Zhang et al., 2017). Therefore, arbitrary ISSR markers are employed for genotyping a polyploidy species like quinoa. Abd El-Moneim et al. (2021) delineated the highest polymorphisms achived through ISSR markes in quinoa compared to other DNA markers.

Moreover, population structure analysis was performed using STRUCTURE software (Pritchard et al., 2000), which inferred quinoa accessions into three main subpopulations and admixture. A previous study was conducted on 96 quinoa accessions collected from six countries, divided into two main populations. Population A comprised accessions mainly from Peru, Bolivia, and Ecuador origins, reflecting that these accessions are genetically related, while population B comprised accessions mainly from Chile and the USA (Barut et al., 2020). PCoA was also performed, and their results showed a strong concordance with the UPGMA cluster analysis. Studies on the genetic diversity of *Chenopodium* species using SSR markers have revealed higher heterozygosity, which could be related to the markers’ nature, the genome’s coverage, and reproductive factors (such as self- and cross-pollination, seed dispersal, and the exchange of genetic information between wild and ancestral relatives) that subjected these species to their native habitats (Costa Tártara et al., 2012; Suresh et al., 2014; Fernández and Noelia, 2015). Previously, several research studies have demonstrated the population structure and diversity of quinoa using genetic markers (Christensen et al., 2007; Fuentes et al., 2009; Zhang et al., 2017).

Genome-wide association study (GWAS) has emerged as an effective tool for simultaneously identifying marker loci linked to desired phenotypic traits in a population that could be valuable for marker-assisted breeding programs (Liu et al., 2018). In the current study, association analysis was performed to assess the associations among ISSR marker loci and agro-morphological traits. Before the GWAS analysis, LD relied on *r*^2^ values, and its critical threshold value was measured to correspond to the 95th percentile of the coefficient square (*r*^2^= 0.12). The LD decay value at the intersection of the LD curve and *r*^2^ threshold was attained at 5 bp. The previously reported LD decay in quinoa was faster (32.4 kb) than that in other crops, including soybean (150 kb), pigeon pea (70 kb), and rapeseed (465 kb), which could be due to the short breeding history and limited selection intensity (Zhou et al., 2015; Varshney et al., 2017; Wu et al., 2019; Patiranage et al., 2022). For GWAS analysis, in total, 99 significant (*p* ≤ 0.01) marker-trait associations (MTAs) were found for 18 phenotypic traits using the GLM method. The number of MTAs ranged from 4 (PD) to 16 (PS) for qualitative traits and from 1 (DM) to 3 (PH) for quantitative traits. Moreover, 72 and 19 significant marker-trait associations (MTAs) were found at significance levels of *p* ≤ 0.05 and *p* ≤ 0.01, respectively, using the MLM method. The number of MTAs ranged from 2 (LM and PD) to 16 (PS) at significance level *p* ≤ 0.05 and one (SC and LS) at significance level *p* ≤ 0.01 for qualitative traits, while it ranged from 1 (SD) to 3 (PH, PL, NOP, P) at significance level *p* ≤ 0.05, and no marker was found to be significant at *p* ≤ 0.01 for quantitative traits using the MLM method. Previously, no genome-wide study has been reported in quinoa using ISSR markers; however, these markers have been employed in various other crops for association mapping (Mohamed et al., 2020; Sonu, 2020). Further, several other molecular markers, including SNPs, have been employed in quinoa for association mapping for agronomically significant traits (Maldonado-Taipe et al., 2022; Patiranage et al., 2022; Nepal et al., 2023).

## Conclusion

A panel of 72 quinoa plants was phenotyped for agro morphological attributes and association-mapping for distinct imperative agronomic traits. The phenotypic data and analysis showed strong correlation of panicle shape, color, density and leafiness (panicle architecture) with yield and productivity in quinoa. The accession SD-121-07 exhibited highest yield per plant possessing green, glomerulate, compact panicle with less leaves followed by accessions S128-04, S33-09, and S470-02 with non-significant difference in yield having green, intermediate, intermediate panicles with less leaves. It showed that change in panicle shape and density from glomerulate shape, compact density panicle to intermediate shape, intermediate density panicles resulting in yield reduction. The accessions SJrecm-03 and SJrecm-05 produced less yield and both exhibited pink, intermediate shape, intermediate density panicles with less leaves. In addition to this, the results also inferred that the accessions with panicle shape amarantiform exhibited less yield. Moreover, a genome-wide association study unraveled the associations between ISSR makers and agro-morphological traits. MLM analysis yielded nine markers associated with eight traits at *p* ≤ 0.01. Similarly, ISSR markers significantly associated with panicle shape and leafiness were also associated with yield per plant. These findings contribute to the provision of authenticity for marker-assisted selection that ultimately would support quinoa breeding programs in future.

## Data availability statement

The original contributions presented in this study are included in this article/Supplementary material, further inquiries can be directed to the corresponding author.

## Author contributions

ZH: Data curation, Formal analysis, Investigation, Methodology, Software, Writing – original draft. SI: Conceptualization, Data curation, Formal analysis, Funding acquisition, Investigation, Methodology, Project administration, Resources, Software, Supervision, Validation, Visualization, Writing – original draft, Writing – review & editing. IH: Conceptualization, Data curation, Formal analysis, Funding acquisition, Investigation, Methodology, Project administration, Resources, Software, Validation, Visualization, Writing – original draft, Writing – review & editing. AH: Software, Visualization, Writing – review & editing. GA-Q: Software, Visualization, Writing – review & editing. EFA: Funding acquisition, Resources, Writing – review & editing. NK: Visualization, Writing – review & editing.
