# Supplementary material for: Empirical phenotyping and genome-wide association study reveal the association of panicle architecture with yield in Chenopodium quinoa
Source: Front Microbiol. 2024 Mar 18;15:1349239. doi: 10.3389/fmicb.2024.1349239 (PMC10982352; doi:10.3389/fmicb.2024.1349239)
Supplement: Supplementary file 1 [file Data_Sheet_1.docx]

**Supplementary Information**

**Supplementary Table S1.** List of forty-three quinoa germplasm imported from King Abdullah University Science Technology (KAUST) were collected from Department of Agronomy University of Agriculture Faisalabad, Pakistan.

| **Sr. #** | **Accessions** | **Origin** | **Sr. #** | **Accessions** | **Origin** |
| --- | --- | --- | --- | --- | --- |
| 1 | D-11973 | Peru | 22 | CHEN-342 | Peru |
| 2 | D-12220 | Peru | 23 | CHEN-391 | Peru |
| 3 | CHEN-470 | Peru | 24 | Javi | Chile |
| 4 | RU-5 | UK | 25 | Salcedo-Inia | ^__^ |
| 5 | PI-614882 | Chile | 26 | PI-614885 | Chile |
| 6 | Ames-13745 | USA | 27 | BO-16 | Chile |
| 7 | Ames-13760 | USA | 28 | BO-163 | ^__^ |
| 8 | PUC-mix-red | Chile | 29 | PI-510551 | Peru |
| 9 | PI-634925 | ^__^ | 30 | CHEN-128 | Bolivia |
| 10 | PI-634919 | Bolivia | 31 | Ames-13748 | USA |
| 11 | D-12377 | Peru | 32 | CHEN-119 | ^__^ |
| 12 | PI-510532 | Peru | 33 | PI-614920 | Bolivia |
| 13 | PI-614927 | Bolivia | 34 | PI-634924 | Chile |
| 14 | Ames-13740 | USA | 35 | D-12258 | Peru |
| 15 | D-12002 | ^__^ | 36 | D-12014 | ^__^ |
| 16 | PI-565283 | ^__^ | 37 | Ames-13751 | USA |
| 17 | CHEN-71 | Bolivia | 38 | CHEN-414 | ^__^ |
| 18 | CHEN-297 | Peru | 39 | CHEN-425 | Peru |
| 19 | CHEN-33 | Bolivia | 40 | CHEN-159 | Bolivia |
| 20 | Ames-13731 | USA | 41 | CHEN-356 | ^__^ |
| 21 | D-12175 | Peru | 42 | D-12166 | Bolivia |

**Supplementary Table S2**. The nucleotide sequence of twenty selected ISSR primers employed in genetic diversity analysis.

| **Sr. #** | **ISSR primers** | **Primer Sequence (5′–3′)** |
| --- | --- | --- |
| 1 | ISSR-1 | (AG)_8_T |
| 2 | ISSR-2 | (GA)_8_T |
| 3 | ISSR-3 | (GA)_8_A |
| 4 | ISSR-4 | (CT)_8_G |
| 5 | ISSR-5 | (TC)_8_G |
| 6 | ISSR-6 | (AC)_8_T |
| 7 | ISSR-7 | (AC)_8_G |
| 8 | ISSR-8 | (GA)_8_TT |
| 9 | ISSR-9 | (AC)_8_YG |
| 10 | ISSR-10 | CGA(AG)_7_ |
| 11 | ISSR-11 | GAC(GA)_7_ |
| 12 | ISSR-12 | GCA(GT)_7_ |
| 13 | ISSR-13 | GCA(TG)_7_ |
| 14 | ISSR-14 | (ATG)_6_ |
| 15 | ISSR-15 | GGG(TGGGG)_2_TG |
| 16 | ISSR-16 | (TC)_8_C |
| 17 | ISSR-17 | (AC)_8_C |
| 18 | ISSR-18 | (GA)_8_CC |
| 19 | ISSR-19 | (CT)_8_AGG |
| 20 | ISSR-20 | (AC)_8_TT |

**Supplementary Table S3.** List of selected plants from each accession Based on scored homogeneity.

| **Plots** | **Accessions** | **Plants Codes** | **No. of plants selected** | **Homogeneity** |
| --- | --- | --- | --- | --- |
| Plot-1 | CHEN-425 | S425-01 | 02 | 95% |
|  |  | S425-02 |  |  |
| Plot-2 | D-12175 | SD121-01 | 10 | 75% |
|  |  | SD121-02 |  |  |
|  |  | SD121-03 |  |  |
|  |  | SD121-04 |  |  |
|  |  | SD121-05 |  |  |
|  |  | SD121-06 |  |  |
|  |  | SD121-07 |  |  |
|  |  | SD121-08 |  |  |
|  |  | SD121-09 |  |  |
|  |  | SD121-10 |  |  |
| Plot-3 | CHEN-391 | S391-01 | 01 | 99% |
| Plot-4 | D-12220 | SD122-01 | 06 | 85% |
|  |  | SD122-02 |  |  |
|  |  | SD122-03 |  |  |
|  |  | SD122-04 |  |  |
|  |  | SD122-05 |  |  |
|  |  | SD122-06 |  |  |
| Plot-5 | PI-614927 | SPrecm-01 | 07 | 75% |
|  |  | SPrecm-02 |  |  |
|  |  | SPrecm-03 |  |  |
|  |  | SPrecm-04 |  |  |
|  |  | SPrecm-05 |  |  |
|  |  | SPrecm-06 |  |  |
|  |  | SPrecm-07 |  |  |
| Plot-6 | CHEN-297 | S297-01 | 04 | 90% |
|  |  | S297-02 |  |  |
|  |  | S297-03 |  |  |
|  |  | S297-04 |  |  |
| Plot-7 | CHEN-128 | S128-01 | 10 | 70% |
|  |  | S128-02 |  |  |
|  |  | S128-03 |  |  |
|  |  | S128-04 |  |  |
|  |  | S128-05 |  |  |
|  |  | S128-06 |  |  |
|  |  | S128-07 |  |  |
|  |  | S128-08 |  |  |
|  |  | S128-09 |  |  |
|  |  | S128-10 |  |  |
| Plot-8 | CHEN-33 | S33-01 | 10 | 70% |
|  |  | S33-02 |  |  |
|  |  | S33-03 |  |  |
|  |  | S33-04 |  |  |
|  |  | S33-05 |  |  |
|  |  | S33-06 |  |  |
|  |  | S33-07 |  |  |
|  |  | S33-08 |  |  |
|  |  | S33-09 |  |  |
|  |  | S33-10 |  |  |
| Plot-9 | CHEN-470 | S470-01 | 10 | 70% |
|  |  | S470-02 |  |  |
|  |  | S470-03 |  |  |
|  |  | S470-04 |  |  |
|  |  | S470-05 |  |  |
|  |  | S470-06 |  |  |
|  |  | S470-07 |  |  |
|  |  | S470-08 |  |  |
|  |  | S470-09 |  |  |
|  |  | S470-10 |  |  |
| Plot-10 | RU-5 Plot | SRUrecm-01 | 02 | 95% |
|  |  | SRUrecm-02 |  |  |
| Plot-11 | PUC-mix-red | SPMrecm-01 | 04 | 90% |
|  |  | SPMrecm-02 |  |  |
|  |  | SPMrecm-03 |  |  |
|  |  | SPMrecm-04 |  |  |
| Plot-12 | JAVI | SJrecm-01 | 07 | 75% |
|  |  | SJrecm-02 |  |  |
|  |  | SJrecm-03 |  |  |
|  |  | SJrecm-04 |  |  |
|  |  | SJrecm-05 |  |  |
|  |  | SJrecm-06 |  |  |
|  |  | SJrecm-07 |  |  |
| Plot-13 | Ames-13760 | SArecm-01 | 02 | 95% |
|  |  | SArecm-02 |  |  |
| Plot-14 | CHEN-71 | S71-01 | 07 | 75% |
|  |  | S71-02 |  |  |
|  |  | S71-03 |  |  |
|  |  | S71-04 |  |  |
|  |  | S71-05 |  |  |
|  |  | S71-06 |  |  |
|  |  | S71-07 |  |  |
| Plot-15 | D-12014 | Excluded from the experimentation | | >50% |
| Plot-16 | PI-614885 |  |  |  |
| Plot-17 | PI-614882 |  |  |  |
| Plot-18 | Ames-13731 |  |  |  |

**Supplementary Table S4.**  Comparison of eigenvalues and variance of proportion (%) of ten principal components of quantitative traits in seventy-two quinoa plants

| **Quantitative traits** | **PC1** | **PC2** | **PC3** | **PC4** | **PC5** | **PC6** | **PC7** | **PC8** | **PC9** | **PC10** |
| --- | --- | --- | --- | --- | --- | --- | --- | --- | --- | --- |
| **NOP** | 0.220 | 0.580 | 0.028 | 0.180 | -0.294 | 0.016 | -0.081 | 0.050 | 0.130 | 0.685 |
| **NOB** | 0.232 | 0.590 | 0.060 | 0.144 | -0.245 | -0.008 | -0.036 | 0.017 | -0.090 | -0.709 |
| **DM** | 0.544 | -0.077 | -0.229 | 0.136 | 0.254 | -0.059 | 0.057 | -0.696 | 0.267 | -0.018 |
| **DF** | 0.495 | -0.051 | -0.219 | 0.188 | 0.389 | -0.264 | 0.204 | 0.601 | -0.209 | 0.037 |
| **PH** | 0.435 | -0.217 | -0.055 | -0.376 | -0.164 | 0.226 | -0.717 | 0.157 | -0.038 | -0.020 |
| **PcL** | 0.282 | -0.037 | 0.037 | -0.654 | -0.366 | -0.035 | 0.593 | -0.012 | -0.038 | 0.033 |
| **SW** | 0.030 | 0.312 | 0.221 | -0.246 | 0.580 | 0.662 | 0.119 | 0.045 | 0.033 | 0.020 |
| **SD** | -0.101 | 0.305 | 0.228 | -0.442 | 0.370 | -0.646 | -0.255 | -0.140 | -0.087 | 0.051 |
| **Y** | -0.220 | 0.180 | -0.609 | -0.234 | 0.051 | -0.044 | -0.023 | 0.235 | 0.648 | -0.115 |
| **P** | -0.169 | 0.186 | -0.647 | -0.115 | 0.016 | 0.129 | -0.029 | -0.223 | -0.655 | 0.094 |
| **Eigenvalue** | 2.347 | 2.197 | 1.819 | 1.244 | 0.995 | 0.637 | 0.400 | 0.197 | 0.135 | 0.027 |
| **Variance (%)** | 23.47 | 21.97 | 18.19 | 12.44 | 9.947 | 6.373 | 4.005 | 1.975 | 1.350 | 0.270 |
| **Cumulative variance** | 23.47 | 45.44 | 63.64 | 76.08 | 86.03 | 92.40 | 96.41 | 98.38 | 99.73 | 100.0 |

**Supplementary Table S5. Correlation between panicle color, shape, leafiness, and density with yield (g).**

| **Quinoa plants** | **Panicle color** | **Panicle shape** | **Panicle leafiness** | **Panicle density** | **Yield** |
| --- | --- | --- | --- | --- | --- |
| **S71-03** | Green | Intermediate | Median | Intermediate | 26.63 |
| **SPrecm-02** | Pink | Intermediate | Less | Compact | 27.64 |
| **SPMrecm-03** | Green with purple | Intermediate | Less | Compact | 22.31 |
| **S470-03** | Green | Intermediate | Less | Compact | 10.42 |
| **S391-01** | Green | Glomerulate | Median | Compact | 9.27 |
| **SPrecm-04** | Yellow | Intermediate | Less | Compact | 21.89 |
| **SD122-02** | Beige | Glomerulate | Less | Intermediate | 18.13 |
| **S470-04** | Green | Intermediate | Less | Compact | 22.45 |
| **SD122-04** | Yellow | Glomerulate | Less | Intermediate | 24.23 |
| **S128-01** | Green | Intermediate | Less | Intermediate | 9.02 |
| **SD121-05** | Green | Glomerulate | Less | Compact | 5.49 |
| **SD122-05** | Yellow | Glomerulate | Median | Intermediate | 17.82 |
| **S128-02** | Green | Intermediate | Less | Intermediate | 24.25 |
| **S71-04** | Green | Intermediate | Median | Intermediate | 22.87 |
| **S128-03** | Green | Intermediate | Less | Intermediate | 6.32 |
| **SD121-06** | Green | Glomerulate | Less | Compact | 14.64 |
| **SD121-07** | Green | Glomerulate | Less | Compact | 42.72 |
| **S297-02** | Green | Intermediate | Less | Lax | 13.01 |
| **SD122-03** | Pink | Glomerulate | Less | Intermediate | 17.32 |
| **SJrecm-02** | Pink | Intermediate | Less | Intermediate | 24.21 |
| **Sprecm-01** | Pink | Glomerulate | Less | Compact | 28.43 |
| **SPrecm-05** | Yellow | Intermediate | Less | Compact | 31.28 |
| **SD121-04** | Green | Glomerulate | Less | Lax | 22.76 |
| **Sprecm-03** | Yellow | Glomerulate | Less | Compact | 19.53 |
| **SPrecm-06** | Yellow | Glomerulate | More | Compact | 20.99 |
| **S128-04** | Green | Intermediate | Less | Intermediate | 36.88 |
| **S128-05** | Green | Intermediate | Less | Intermediate | 20.64 |
| **SJrecm-01** | Pink | Intermediate | More | Intermediate | 13.24 |
| **SJrecm-03** | Pink | Intermediate | Less | Intermediate | 5.68 |
| **S128-06** | Green | Intermediate | Less | Intermediate | 17.7 |
| **S470-08** | Yellow | Intermediate | Less | Intermediate | 26.89 |
| **S470-09** | Yellow | Intermediate | Less | Intermediate | 15.95 |
| **S128-07** | Green | Intermediate | Less | Intermediate | 10.87 |
| **SD121-08** | Green | Glomerulate | Less | Compact | 26.38 |
| **SD121-09** | Green | Glomerulate | Less | Compact | 26.63 |
| **SPrecm-07** | Yellow | Glomerulate | More | Compact | 22.32 |
| **SD121-10** | Green | Glomerulate | Less | Compact | 18.62 |
| **S470-05** | Green | Intermediate | Less | Compact | 10.28 |
| **S128-08** | Green | Intermediate | Less | Intermediate | 9.08 |
| **S128-09** | Green | Intermediate | Less | Intermediate | 12.32 |
| **S128-10** | Green | Intermediate | Less | Intermediate | 14.31 |
| **S33-01** | Green | Intermediate | Less | Intermediate | 15.89 |
| **S425-02** | Pink | Amaranthiform | Median | Intermediate | 22.29 |
| **SRUrecm-01** | Green | Amaranthiform | Less | Intermediate | 19.61 |
| **S71-05** | Green | Intermediate | Median | Intermediate | 19.08 |
| **S33-02** | Green | Intermediate | Less | Intermediate | 9.48 |
| **SJrecm-04** | Pink | Intermediate | Less | Intermediate | 24.27 |
| **SJrecm-06** | Pink | Intermediate | Median | Intermediate | 14.95 |
| **S33-03** | Green | Intermediate | Less | Intermediate | 13.1 |
| **SJrecm-07** | Pink | Intermediate | Median | Intermediate | 23.76 |
| **S71-01** | Green with purple | Intermediate | Median | Intermediate | 10.81 |
| **S71-06** | Green | Intermediate | Median | Intermediate | 9.76 |
| **S425-01** | Pink | Amaranthiform | Median | Intermediate | 9.98 |
| **S470-10** | Yellow | Intermediate | Less | Intermediate | 17.01 |
| **S33-04** | Green | Intermediate | Less | Intermediate | 11.23 |
| **S470-07** | Beige | Intermediate | Less | Intermediate | 21.29 |
| **S33-05** | Green | Intermediate | Less | Intermediate | 8.5 |
| **S71-02** | Dark colored | Intermediate | Median | Intermediate | 14.09 |
| **S33-06** | Green | Intermediate | Less | Intermediate | 12.6 |
| **SJrecm-05** | Pink | Intermediate | Less | Intermediate | 5.21 |
| **S33-07** | Green | Intermediate | Less | Intermediate | 16.22 |
| **S71-07** | Green | Intermediate | Median | Intermediate | 12.47 |
| **SPMrecm-04** | Green with purple | Intermediate | Less | Intermediate | 34.24 |
| **SRUrecm-02** | Green | Amaranthiform | Less | Intermediate | 7.88 |
| **S33-08** | Green | Intermediate | Less | Intermediate | 7.32 |
| **S33-09** | Green | Intermediate | Less | Intermediate | 36.24 |
| **S33-10** | Green | Intermediate | Less | Intermediate | 24.97 |
| **S470-01** | Green | Intermediate | Less | Intermediate | 25.57 |
| **S470-02** | Green | Intermediate | Less | Intermediate | 34.44 |
| **SPMrecm-01** | Green with purple | Intermediate | Less | Compact | 10.71 |
| **SPMrecm-02** | Green with purple | Intermediate | Less | Compact | 22.83 |
| **SArecm-01** | Green | Intermediate | Median | Compact | 9.66 |

**Supplementary Table S6.** Genetic diversity parameters of twenty ISSR markers employed in genetic diversity analysis.

| Sr. # | **Primer codes** | **Major Allele Frequency** | **Gene Diversity** | **PIC** |
| --- | --- | --- | --- | --- |
| 1 | ISSR-1 | 0.7037 | **0.3977** | **0.3159** |
| 2 | ISSR-2 | 0.7037 | 0.3977 | 0.3159 |
| **3** | ISSR-3 | 0.6806 | 0.4226 | 0.3309 |
| **4** | ISSR-4 | 0.6806 | 0.4226 | 0.3309 |
| **5** | ISSR-5 | 0.6505 | 0.4225 | 0.3283 |
| **6** | ISSR-6 | 0.6458 | 0.4229 | 0.2729 |
| **7** | ISSR-7 | 0.5230 | 0.4273 | 0.3286 |
| **8** | ISSR-8 | 0.6389 | 0.4304 | 0.3359 |
| **9** | ISSR-9 | 0.6296 | 0.4527 | 0.3486 |
| **10** | ISSR-10 | 0.6273 | 0.4556 | 0.3510 |
| **11** | ISSR-11 | 0.6620 | 0.4308 | 0.3356 |
| **12** | ISSR-12 | 0.6458 | 0.4484 | 0.3471 |
| **13** | ISSR-13 | 0.6111 | 0.4613 | 0.3540 |
| **14** | ISSR-14 | 0.6111 | 0.4613 | 0.3540 |
| **15** | ISSR-15 | 0.6435 | 0.4506 | 0.3485 |
| **16** | ISSR-16 | 0.6435 | 0.4506 | 0.3485 |
| **17** | ISSR-17 | 0.6111 | 0.4694 | 0.3589 |
| **18** | ISSR-18 | 0.6111 | 0.4694 | 0.3589 |
| **19** | ISSR-19 | 0.6458 | 0.4288 | 0.3304 |
| **20** | ISSR-20 | 0.6458 | 0.4288 | 0.3304 |

**Supplementary Table S8.** Classification of quinoa plants and their inferred subpopulation identities.

| **No.** | **Accessions** | **Inferred Subpopulations** | **Population ID** |
| --- | --- | --- | --- |
| 1 | S425-01 | Admixture | 1 |
| 2 | S425-02 | Admixture | 2 |
| 3 | SD121-04 | 3 | 3 |
| 4 | SD121-05 | 3 | 4 |
| 5 | SD121-06 | 3 | 5 |
| 6 | SD121-07 | 3 | 6 |
| 7 | SD121-08 | 3 | 7 |
| 8 | SD121-09 | 3 | 8 |
| 9 | SD121-10 | 3 | 9 |
| 10 | S391-01 | Admixture | 10 |
| 11 | SD122-02 | 2 | 11 |
| 12 | SD122-03 | 2 | 12 |
| 13 | SD122-04 | 2 | 13 |
| 14 | SD122-05 | 2 | 14 |
| 15 | SPrecm-01 | 2 | 15 |
| 16 | SPrecm-02 | 2 | 16 |
| 17 | SPrecm-03 | 2 | 17 |
| 18 | SPrecm-04 | 2 | 18 |
| 19 | SPrecm-05 | 2 | 19 |
| 20 | SPrecm-06 | 2 | 20 |
| 21 | SPrecm-07 | 2 | 21 |
| 22 | S297-02 | 2 | 22 |
| 23 | S128-01 | 1 | 23 |
| 24 | S128-02 | 1 | 24 |
| 25 | S128-03 | 1 | 25 |
| 26 | S128-04 | 1 | 26 |
| 27 | S128-05 | 1 | 27 |
| 28 | S128-06 | 1 | 28 |
| 29 | S128-07 | 1 | 29 |
| 30 | S128-08 | 1 | 30 |
| 31 | S128-09 | 1 | 31 |
| 32 | S128-10 | 1 | 32 |
| 33 | S33-01 | 1 | 33 |
| 34 | S33-02 | 1 | 34 |
| 35 | S33-03 | 1 | 35 |
| 36 | S33-04 | 1 | 36 |
| 37 | S33-05 | 1 | 37 |
| 38 | S33-06 | 1 | 38 |
| 39 | S33-07 | 1 | 39 |
| 40 | S33-08 | 1 | 40 |
| 41 | S33-09 | 1 | 41 |
| 42 | S33-10 | 1 | 42 |
| 43 | S470-01 | 3 | 43 |
| 44 | S470-02 | 3 | 44 |
| 45 | S470-03 | 3 | 45 |
| 46 | S470-04 | 3 | 46 |
| 47 | S470-05 | 3 | 47 |
| 48 | S470-07 | 3 | 48 |
| 49 | S470-08 | 3 | 49 |
| 50 | S470-09 | 3 | 50 |
| 51 | S470-10 | 3 | 51 |
| 52 | SRUrecm-01 | 2 | 52 |
| 53 | SRUrecm-02 | 2 | 53 |
| 54 | SPMrecm-01 | 2 | 54 |
| 55 | SPMrecm-02 | 2 | 55 |
| 56 | SPMrecm-03 | 2 | 56 |
| 57 | SPMrecm-04 | 2 | 57 |
| 58 | SJrecm-01 | 2 | 58 |
| 59 | SJrecm-02 | 2 | 59 |
| 60 | SJrecm-03 | 2 | 60 |
| 61 | SJrecm-04 | 2 | 61 |
| 62 | SJrecm-05 | 2 | 62 |
| 63 | SJrecm-06 | 2 | 63 |
| 64 | SJrecm-07 | 2 | 64 |
| 65 | SArecm-01 | Admixture | 65 |
| 66 | S71-01 | 1 | 66 |
| 67 | S71-02 | 1 | 67 |
| 68 | S71-03 | 1 | 68 |
| 69 | S71-04 | 1 | 69 |
| 70 | S71-05 | 1 | 70 |
| 71 | S71-06 | 1 | 71 |
| 72 | S71-07 | 1 | 72 |

**Supplementary Table S9.** Linkage disequilibrium estimations.

| Total number of plants | 72 | |
| --- | --- | --- |
| Total number of markers | 20 | |
| Pairwise estimation | N | % |
| r^2^ < 0.2 | 295 | 4.1 |
| 0.3 > r^2^ > 0.2 | 199 | 2.7 |
| 0.5 > r^2^ > 0.3 | 113 | 1.5 |
| r^2^ > 0.5 | 20 | 0.3 |
| Total pairwise combinations | 7140 | 100 |

**Supplementary Table S10.** ISSR markers associated with qualitative and quantitative traits in quinoa plants at significance level p ≤ 0.01 and phenotypic variance (R^2^) under GLM analysis.

| **Trait** | **Marker** | **p ≤ 0.01** | **R^2^** |
| --- | --- | --- | --- |
| **Panicle color** | ISSR-1 | 0.00404 | 0.10483 |
|  | ISSR-2 | 0.00404 | 0.10483 |
|  | ISSR-3 | 0.000369 | 0.15555 |
|  | ISSR-4 | 0.000369 | 0.15555 |
|  | ISSR-7 | 0.00000415 | 0.24405 |
|  | ISSR-8 | 0.0000172 | 0.21704 |
|  | ISSR-11 | 0.00152 | 0.12581 |
|  | ISSR-12 | 0.00152 | 0.12581 |
|  | ISSR-19 | 0.00597 | 0.09637 |
|  | ISSR-20 | 0.00597 | 0.09637 |
| **Stem color** | ISSR-5 | 0.000217 | 0.14228 |
|  | ISSR-6 | 0.000217 | 0.14228 |
|  | ISSR-10 | 0.0000433 | 0.17001 |
|  | ISSR-11 | 0.00545 | 0.08402 |
|  | ISSR-12 | 0.00545 | 0.08402 |
|  | ISSR-13 | 0.00345 | 0.09244 |
|  | ISSR-14 | 0.00345 | 0.09244 |
|  | ISSR-15 | 0.0019 | 0.10341 |
|  | ISSR-16 | 0.0019 | 0.10341 |
|  | ISSR-17 | 0.0000841 | 0.1587 |
|  | ISSR-18 | 0.0000841 | 0.1587 |
|  | ISSR-19 | 0.0000964 | 0.15637 |
|  | ISSR-20 | 0.0000964 | 0.15637 |
| **Panicle shape** | ISSR-1 | 0.00268 | 0.11085 |
|  | ISSR-2 | 0.00268 | 0.11085 |
|  | ISSR-3 | 0.000432 | 0.14849 |
|  | ISSR-4 | 0.000432 | 0.14849 |
|  | ISSR-6 | 0.00155 | 0.12228 |
|  | ISSR-7 | 0.00428 | 0.10098 |
|  | ISSR-8 | 0.000482 | 0.14627 |
|  | ISSR-9 | 1.57E-10 | 0.39917 |
|  | ISSR-10 | 1.57E-10 | 0.39917 |
|  | ISSR-11 | 0.00231 | 0.11389 |
|  | ISSR-12 | 0.00231 | 0.11389 |
|  | ISSR-13 | 8.61E-06 | 0.22455 |
|  | ISSR-14 | 8.61E-06 | 0.22455 |
|  | ISSR-15 | 0.00707 | 0.09039 |
|  | ISSR-16 | 0.00707 | 0.09039 |
|  | ISSR-17 | 0.00201 | 0.11685 |
|  | ISSR-18 | 0.00201 | 0.11685 |
| **Panicle length** | ISSR-1 | 0.0000192 | 0.23052 |
|  | ISSR-2 | 0.0000192 | 0.23052 |
|  | ISSR-3 | 0.000121 | 0.19144 |
|  | ISSR-4 | 0.000121 | 0.19144 |
|  | ISSR-7 | 0.000582 | 0.15667 |
|  | ISSR-8 | 0.000791 | 0.14975 |
|  | ISSR-9 | 0.00544 | 0.10555 |
|  | ISSR-11 | 0.00442 | 0.11032 |
|  | ISSR-12 | 0.00442 | 0.11032 |
|  | ISSR-13 | 0.00141 | 0.13667 |
|  | ISSR-14 | 0.00141 | 0.13667 |
|  | ISSR-15 | 0.00303 | 0.1191 |
|  | ISSR-16 | 0.00303 | 0.1191 |
|  | ISSR-17 | 0.0000159 | 0.23443 |
|  | ISSR-18 | 0.0000159 | 0.23443 |
| **Panicle density** | ISSR-8 | 0.00187 | 0.11745 |
|  | ISSR-11 | 0.000235 | 0.15951 |
|  | ISSR-12 | 0.000235 | 0.15951 |
| **Leaf shape** | ISSR-3 | 0.000000719 | 0.30709 |
|  | ISSR-4 | 0.000000719 | 0.30709 |
|  | ISSR-7 | 0.00252 | 0.1279 |
|  | ISSR-9 | 0.00566 | 0.10852 |
|  | ISSR-11 | 0.0000531 | 0.31303 |
|  | ISSR-12 | 0.0000531 | 0.31303 |
| **Leaf granules** | ISSR-5 | 0.000858 | 0.14839 |
|  | ISSR-6 | 0.000858 | 0.14839 |
|  | ISSR-7 | 0.000478 | 0.15772 |
|  | ISSR-8 | 0.000843 | 0.11069 |
|  | ISSR-11 | 0.0000304 | 0.16488 |
|  | ISSR-12 | 0.00788 | 0.07233 |
|  | ISSR-13 | 0.000864 | 0.11027 |
|  | ISSR-14 | 0.000864 | 0.11027 |
|  | ISSR-15 | 0.00669 | 0.07519 |
|  | ISSR-16 | 0.00669 | 0.07519 |
|  | ISSR-17 | 0.000150 | 0.17582 |
|  | ISSR-18 | 0.000150 | 0.17582 |
|  | ISSR-19 | 0.000427 | 0.15951 |
|  | ISSR-20 | 0.000427 | 0.15951 |
| **Days to maturity** | ISSR-6 | 0.00734 | 0.10165 |
| **Plant height** | ISSR-7 | 0.00587 | 0.09662 |
|  | ISSR-19 | 0.00637 | 0.09483 |
|  | ISSR-20 | 0.00637 | 0.09483 |
| **Panicle length** | ISSR-19 | 0.0017 | 0.13688 |
|  | ISSR-20 | 0.0017 | 0.13688 |
| **Seed weight** | ISSR-8 | 0.00724 | 0.10018 |
|  | ISSR-17 | 0.005 | 0.10886 |
|  | ISSR-18 | 0.005 | 0.10886 |
| **Productivity** | ISSR-15 | 0.00984 | 0.09339 |
|  | ISSR-16 | 0.00984 | 0.09339 |

**Supplementary Figures**


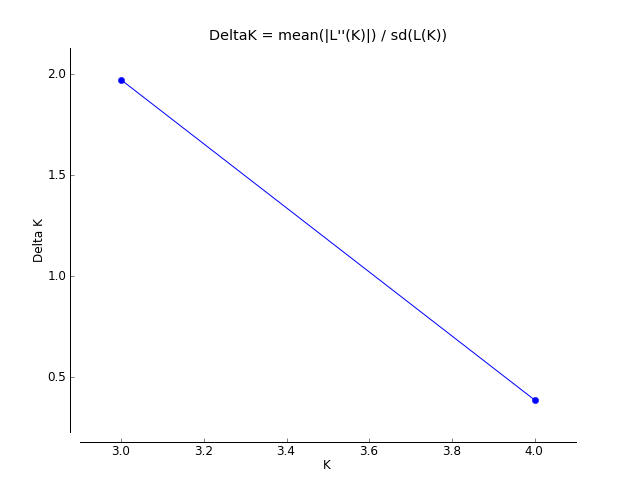


**Supplementary Figure S1.** STRUCTURE HARVESTER results showing the ΔK values for each number of subpopulations (K).
